# The NLRP11 Protein Bridges the Histone Lysine Acetyltransferase KAT7 to Acetylate Vimentin in the Early Stage of Lung Adenocarcinoma

**DOI:** 10.1002/advs.202300971

**Published:** 2023-07-09

**Authors:** Rui Yang, Weilin Peng, Shuai Shi, Xiong Peng, Qidong Cai, Zhenyu Zhao, Boxue He, Guangxu Tu, Wei Yin, Yichuan Chen, Yuqian Zhang, Fang Liu, Xiang Wang, Desheng Xiao, Yongguang Tao

**Affiliations:** ^1^ Department of Pathology Xiangya Hospital and School of Basic Medicine Central South University Changsha Hunan 410008 China; ^2^ NHC Key Laboratory of Carcinogenesis Cancer Research Institute and School of Basic Medicine Central South University Changsha Hunan 410078 China; ^3^ Department of Thoracic Surgery The Second Xiangya Hospital Central South University Changsha Hunan 410011 China; ^4^ Hunan Key Laboratory of Early Diagnosis and Precise Treatment of Lung Cancer the Second Xiangya Hospital of Central South University Changsha Hunan 410011 China; ^5^ Department of Cardiovascular Surgery The Second Xiangya Hospital Central South University Changsha Hunan 410011 China; ^6^ Department of Thoracic Surgery The First Affiliated Hospital School of Medicine Zhejiang University Hangzhou Zhejiang 310000 China; ^7^ Clinic Nursing Teaching and Research Section The Second Xiangya Hospital Central South University Changsha Hunan 410011 China

**Keywords:** epithelial–mesenchymal transition, non‐histone acetylation, tumorigenesis

## Abstract

Accumulation of vimentin is the core event in epithelial–mesenchymal transition (EMT). Post‐translational modifications have been widely reported to play crucial roles in imparting different properties and functions to vimentin. Here, a novel modification of vimentin, acetylated at Lys^104^ (vimentin‐K104Ac) is identified, which is stable in lung adenocarcinoma (LUAD) cells. Mechanistically, NACHT, LRR, and PYD domain‐containing protein 11 (NLRP11), a regulator of the inflammatory response, bind to vimentin and promote vimentin‐K104Ac expression, which is highly expressed in the early stages of LUAD and frequently appears in vimentin‐positive LUAD tissues. In addition, it is observed that an acetyltransferase, lysine acetyltransferase 7 (KAT7), which binds to NLRP11 and vimentin, directly mediates the acetylation of vimentin at Lys^104^ and that the cytoplasmic localization of KAT7 can be induced by NLRP11. Malignant promotion mediated by transfection with vimentin‐K104Q is noticeably greater than that mediated by transfection with vimentin‐WT. Further, suppressing the effects of NLRP11 and KAT7 on vimentin noticeably inhibited the malignant behavior of vimentin‐positive LUAD in vivo and in vitro. In summary, these findings have established a relationship between inflammation and EMT, which is reflected via KAT7‐mediated acetylation of vimentin at Lys^104^ dependent on NLRP11.

## Graphical abstract

Under normal conditions, vimentin is degraded by ubiquitin‐proteasome pathway. In cancer cells persistently expressing NLRP11, KAT7 tends to translocate from the nucleus to the cytoplasm and is recruited to bind with vimentin by NLRP11, resulting in vimentin acetylation at K104 and inhibition of ubiquitin‐independent proteasomal degradation, thereby contributing to the malignant phenotype related to epithelial–mesenchymal transition in LUAD cells.

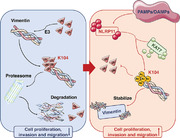

## 1 Introduction

Lung cancer is the leading cause of malignancy‐related deaths worldwide, with an estimated 2.207 million new cases and 1.796 million deaths in 2020. The incidence and mortality of lung cancer increase annually.^[^ ^1^ ^]^ Lung adenocarcinoma (LUAD) is the most common histological subtype, accounting for ≈40% of all lung cancers. Each clinical stage of lung cancer differentiates the overall survival. The 60‐month overall survival rate of lung cancer patients gradually decreased from 92% in stage IA1 to 0% in IVB,^[^ ^2^ ^]^ which reflects the importance of early diagnosis and treatment in improving the prognosis of lung cancer patients. Despite improvements in early diagnosis and individualized treatment, the 5‐year survival rate of patients remains ≈23%, which is mainly because only 21% of cases are diagnosed at an early stage of lung cancer.^[^ ^3^ ^]^ Therefore, uncovering new core molecular network signatures to establish novel diagnostic and treatment models for the early stages of LUAD is essential for reducing LUAD impairment in the population.

Different stages of malignant tumor progression involve extremely complex regulatory networks. Tumor‐promoting inflammation is a prominent constituent of tumor‐enabling characteristics.^[^ ^4^ ^]^ Cytokine disorders, mediated by inflammasomes owing to the activation of pathogen‐/damage‐associated molecular patterns (PAMPs/DAMPs), are a notable manifestation of cancer progression caused by inflammation.^[^ ^5^, ^6^ ^]^ Nucleotide‐binding and oligomerization domain‐like receptors (NLRs) are involved in the composition of inflammasomes and are responsible for recognizing PAMPs/DAMPs in immune and stromal cells to regulate downstream signals.^[^ ^7^, ^8^ ^]^ Various physical and chemical stresses and pathogenic infections are associated with the progression of lung cancer, including smoking and bacterial and viral infections.^[^ ^9^, ^10^ ^]^ However, the relationship between NLRs and cancer remains to be elucidated, which is of vital for revealing their potential value as diagnostic markers and drug targets.

Type‐III intermediate filament (IF) vimentin is ubiquitously expressed in normal mesenchymal cells and various epithelial cancers, and has always been recognized as a marker for epithelial–mesenchymal transition (EMT),^[^ ^11^ ^]^ which is one of the core mechanisms of malignant transformation in epithelial cancers.^[^ ^12^, ^13^ ^]^ Vimentin participates in pathogen internalization and the immune response,^[^ ^14^, ^15^ ^]^ interacts with NACHT, LRR, And PYD Domains‐Containing Protein 3 (NLRP3) and NLR Family, CARD Domain Containing 2, and mediates the activation of inflammatory pathways.^[^ ^16^, ^17^ ^]^ Nevertheless, the interactions among vimentin, NLRs, and molecular event outcomes have not been reported in malignant tumor models. Post‐translational modifications (PTMs), such as phosphorylation, acetylation, and ubiquitination are associated with the functions and stability of vimentin.^[^ ^18^ ^]^ Vimentin phosphorylation at Ser^38^, Ser^39^, Ser^55^, Ser^56^, and Ser^82^ causes dynamic changes in its dissolution, depolymerization, and assembly and participates in the cell cycle, migration, and apoptosis.^[^ ^19^, ^20^, ^21^, ^22^ ^]^ Citrullination enables vimentin to be recognized by the immune system by endowing it with immunogenicity, thereby making it participate in the development of autoimmune diseases and tumors.^[^ ^23^, ^24^, ^25^ ^]^ Ubiquitination at Lys^97^ induces degradation of vimentin through the proteasome pathway.^[^ ^26^ ^]^ Moreover, the acetylation of vimentin at Lys^120^ promotes EMT and cell metastasis.^[^ ^27^, ^28^ ^]^ However, there are few reports on PTMs of vimentin in lung cancer, particularly in the early stages.

In this study, we demonstrated a novel pathway through which NACHT, LRR, And PYD Domains‐Containing Protein 11 (NLRP11) promotes the malignant phenotype of LUAD cells in vitro and in vivo by acetylating vimentin at Lys^104^ (vimentin‐K104Ac), which is mediated by the recruitment of acetyltransferase lysine acetyltransferase 7 (KAT7). It is likely that NLRP11 and vimentin‐K104Ac are potential biomarkers and therapeutic targets for early diagnosis and treatment of LUAD.

## 2 Results

### 2.1 NLRP11 is Highly Expressed in the Early Stage of LUAD and Associated with Poor Prognosis in LUAD Patients

**Figure 1:**
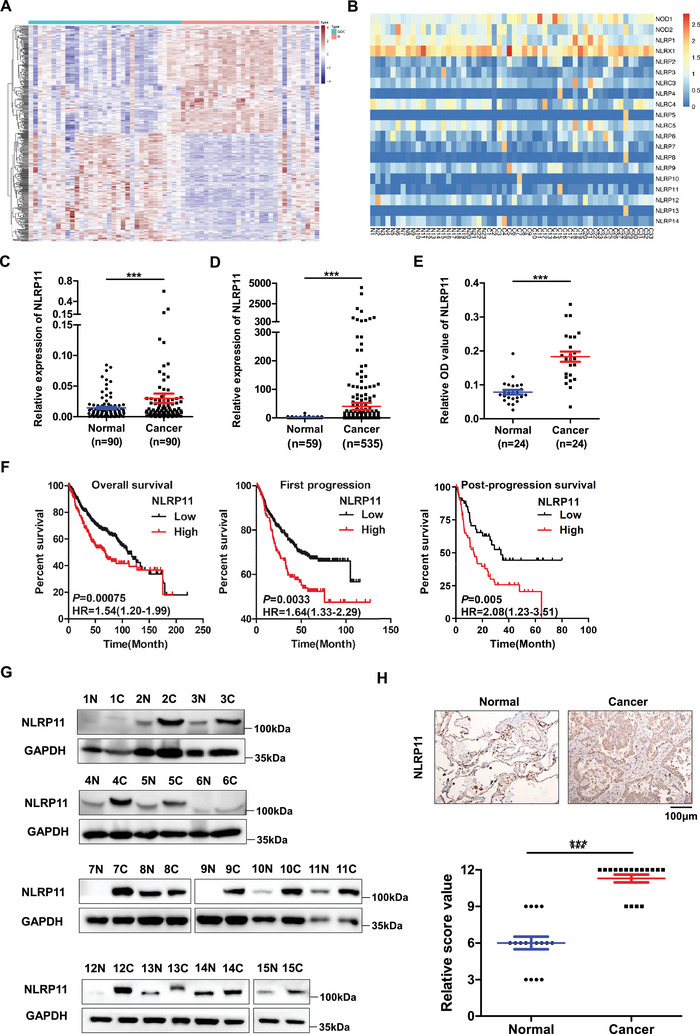
NLRP11 is highly expressed in the early stage of lung adenocarcinoma (LUAD) in tissues and cell lines. A) Heatmap of differentially expressed genes from RNA‐seq in early LUAD tissues and adjacent normal tissues from 23 patients. B) The heatmap shows the differential expression of 20 NLRs in early LUAD tissues and adjacent normal tissues. C) Real‐time quantitative reverse transcriptase (RT‐qPCR) was performed to quantify NLRP11 mRNA in 90 LUAD tissues and corresponding adjacent normal tissues. D) Comparison of NLRP11 mRNA in 59 normal lung tissues and 535 LUAD tissues from the TCGA database. E) ELISA was used to detect the expression of NLRP11 in serum from early LUAD patients (cancer) and the Blood Transfusion Department (Normal). F) The relationships between NLRP11 mRNA expression and the prognosis of LUAD, including overall survival (OS), first progression (FP), and post‐progression survival (PPS), were analyzed in gene chips from the Kaplan–Meier plotter database. G,H) Western blotting and immunohistochemistry (IHC) were used to measure the expression of NLRP11 protein in 15 and 17 pairs of early LUAD and corresponding adjacent normal tissues, respectively (*** *P* < 0.001).

In the present study, differential pathways in gene transcription levels in early LUAD were mainly concentrated in a disordered immune response, including cytokine—cytokine receptor interactions, viral protein interactions with—cytokine receptors, and primary immunodeficiency (**Figure** **1**A; Figure S1A, Supporting Information). Considering the pivotal role of NLRs as cytokine regulators,^[^ ^29^, ^30^ ^]^ we further analyzed the difference in NLR expression between the early LUAD tissues and their corresponding adjacent noncancerous tissues. The results of RNA‐seq and real‐time quantitative reverse transcriptase PCR (RT‐qPCR) demonstrated that the mRNA expression of NLRP7 and NLRP11 increased significantly in LUAD tissues with the filter criteria of a fold‐change ≥3 and a *P* value <0.05, compared to other NLR members (Figure 1B,C; Figure S1B,C, Supporting Information); the expression of NLRP7 and NLRP11 from The Cancer Genome Atlas (TCGA) database (https://portal.gdc.cancer.gov/) exhibited the same trend (Figure 1D; Figure S1D, Supporting Information). However, NLRP11, but not NLRP7, exhibited a close relationship with the poor prognosis of patients with LUAD, according to data from the Kaplan‒Meier Plotter (http://kmplot.com/analysis/) (Figure 1F; Figure S1E, Supporting Information). The Cancer Cell Line Encyclopedia (CCLE, https://sites.broadinstitute.org/ccle) database and RT‐qPCR revealed that NLRP11 mRNA expression was enhanced in lung cancer cell lines compared to that in bronchial epithelial cell lines (Figure S1F,G, Supporting Information). Subsequently, we observed notably high expression of NLRP11 in LUAD cell lines, early LUAD tissues, and the serum of patients with early LUAD using western blotting, immunohistochemistry (IHC), and ELISA (Figure S1H, Supporting Information) (Figure 1E,G,H). These findings indicate that high NLRP11 expression is closely associated with the occurrence and progression of early stage LUAD.

### 2.2 NLRP11 Interacts with Vimentin

**Figure 2:**
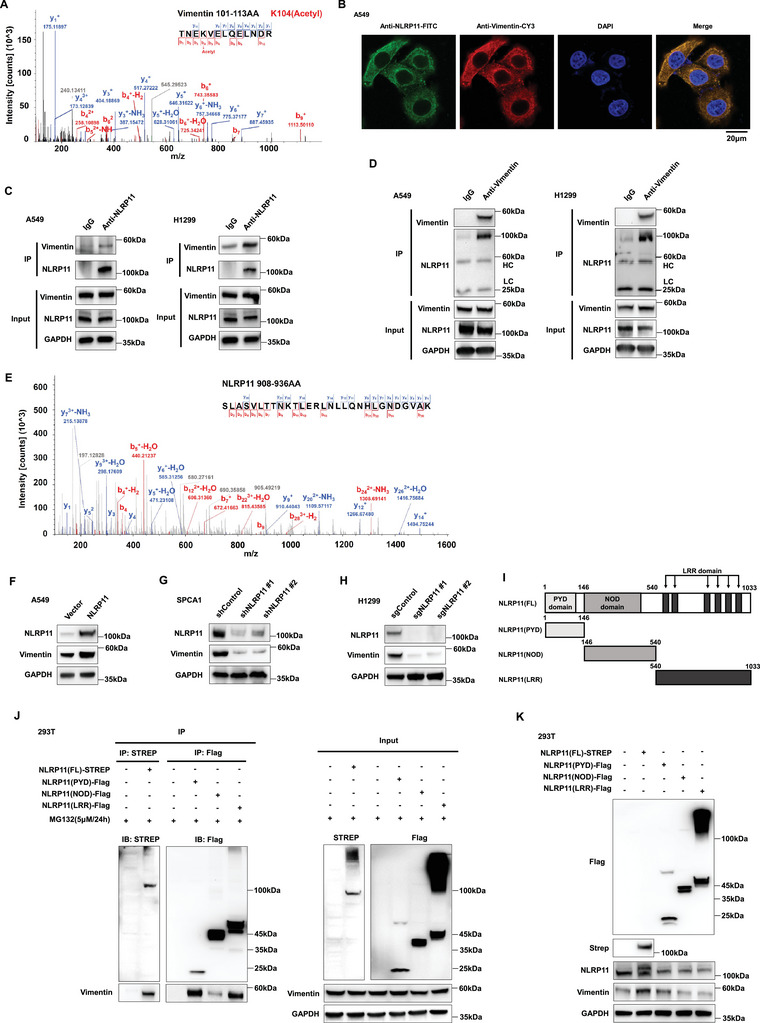
NLRP11 interacted with vimentin. A) LC‒MS/MS was performed to reveal the potentially interacting proteins with NLRP11, and post‐translational modifications (PTMs) of amino acids depended on the mass shift of the peptide segment. The secondary mass spectrogram exhibited the peptide fragment of vimentin‐K104Ac binding to the NLRP11 protein in H1299 cell. B) The colocalization of endogenous NLRP11 and vimentin was demonstrated with immunofluorescence in H1299 cells. C,D) Co‐immunoprecipitation (co‐IP) and western blot analyses of endogenous NLRP11 interacting with vimentin in A549 and H1299 cells. (HC: Heavy chain protein, LC: Light chain protein). E) LC‒MS/MS was performed to detect the interacting proteins with vimentin in H1299 cell. The secondary mass spectrogram exhibited the peptide fragment of NLRP11 binding to the vimentin protein in H1299 cell. F–H) Western blot analysis showed that overexpressing NLRP11 promotes vimentin expression protein in A549 cells (F) and that knocking down and knocking out NLRP11 suppresses vimentin protein expression in SPCA1 (G) and H1299 cells (H). I) Schematic diagram of the Flag‐labeled truncated mutant of NLRP11, and the numbers indicate the amino acid position. J) Co‐IP and western blotting were performed to detect the binding ability of different truncations to vimentin. K) Western blot analysis of the effects of NLRP11 protein truncations on the expression of vimentin.

In previous reports, NLRP11 tended to play a biological role through protein‒protein interactions.^[^ ^31^, ^32^, ^33^ ^]^ To explore the potential mechanism of NLRP11 in LUAD, we used co‐immunoprecipitation (co‐IP) and liquid chromatography tandem mass spectrometry (LC‐MS/MS) to detect the proteins interacting with NLRP11. Meanwhile, we conducted Gene Set Enrichment Analysis (GSEA) on a single gene set in the TCGA database, which contained genes prominently related to the expression of NLRP11 mRNA. The results showed that the pathways positively correlated with NLRP11 were involved in multiple immune responses and cytokine expression (Figure S2A, Supporting Information). Coincidentally, these pathways were consistent with the upregulated pathways in early‐stage LUAD tissues (Figure S1A, Supporting Information), whereas the negatively correlated pathways included ubiquitin‐mediated proteolysis, adherens junctions, and autophagy (Figure S2A,B, Supporting Information). In the follow‐up Gene Ontology (GO) analysis of quantitative proteomics in six pairs of early‐stage LUAD tissues and their adjacent noncancerous tissues, we observed that changes in cell adhesion, viral transcription, and the innate immune response pathway were the most notable protein expression characteristics of early‐stage LUAD (Figure S2C,D, Supporting Information). Joint analysis of NLRP11‐interacting proteins using LC‐MS/MS revealed that NLRP11 binds to the vimentin protein, which is closely related to the cell adhesion pathway (**Figure** **2**A). Confocal microscopy and co‐IP results demonstrated that NLRP11 directly bound to vimentin (Figure 2B–D). Liquid chromatography tandem mass spectrometry (LC‒MS/MS) showed that the peptide segments of NLRP11 also appeared in the vimentin‐interacting protein via co‐IP (Figure 2E). Western blotting results indicated that NLRP11 and vimentin were highly expressed in early‐stage LUAD tissues and cell lines (Figure S3A,B, Supporting Information). In the early stage of LUAD and lymph node metastasis of LUAD tissues, we observed that NLRP11 has always been expressed in tissues with positive vimentin (Figure S3C,D, Supporting Information). To uncover the function and regulation of NLRP11 on vimentin in LUAD, we stably overexpressed NLRP11 in A549 and PC9 cell lines, both of which had lower endogenous expression of NLRP11, and found that the overexpression of NLRP11 in A549 cells promoted the expression of vimentin protein instead of mRNA (Figure 2F) (Figure S3E, Supporting Information). However, NLRP11 did not appreciably upregulate the vimentin protein or mRNA levels in PC9 cells (Figure S3F,G, Supporting Information). We then stably knocked down and knocked out NLRP11 in SPCA1 and H1299 cells, which resulted from higher endogenous expression of NLRP11. Suppression of NLRP11 in SPCA1 and H1299 cells inhibited vimentin protein (Figure 2G,H), but not mRNA, expression (Figure S3I,J, Supporting Information). In addition, ELISA results demonstrated that the overexpression, knockdown, or knockout of NLRP11 did not considerably affect the expression of vimentin in the supernatant of the cell culture medium, indicating that NLRP11 had no remarkable effect on vimentin secretion (Figure S3H,K,L, Supporting Information). Moreover, we found that the PYD and LRR domains of NLRP11 could bind to vimentin (Figure 2I,J). In addition, only the full‐length NLRP11 markedly increased vimentin expression (Figure 2K). Collectively, these results indicate that NLRP11 binds to and upregulates vimentin; however, the underlying mechanism remains unclear.

### 2.3 NLRP11 Promotes Vimentin K104 Acetylation and Maintains the Stability of Vimentin Protein

**Figure 3:**
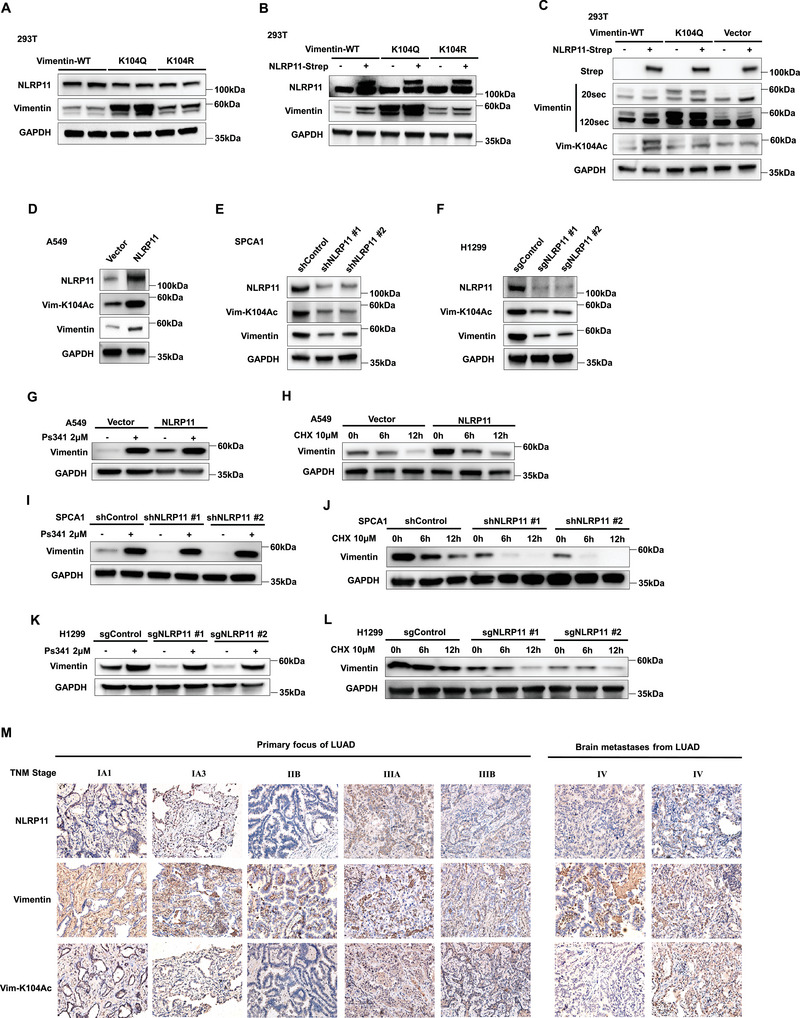
NLRP11 accelerates acetylation of vimentin‐K104 and maintains stability of vimentin. A) The expression of vimentin was detected using western blotting in 293T cells transfected with vimentin‐WT, K104Q, and K104R. B) The regulation of NLRP11 on vimentin protein was tested using western blot in 293T cells transfected with vimentin‐WT, K104Q, and K104R. C) The expression levels of vimentin and vimentin‐K104Ac were measured using western blotting in 293T cells co‐transfected with vimentin‐WT or vimentin‐K104Q and NLRP11. D–F) The expression of vimentin and vimentin‐K104Ac was detected in A549 oeNLRP11 (D), SPCA1 shNLRP11 (E), and H1299 sgNLRP11 (F) cell lines and corresponding control cells. G,I,K) The regulation of NLRP11 on vimentin protein was detected under proteasome inhibition by ps341 (2 µm, 24 h) in the A549 oeNLRP11 (G), SPCA1 shNLRP11 (I) and H1299 sgNLRP11 (K) cell lines and corresponding control cells. H,J,L) The influences of NLRP11 on vimentin protein were detected under interference of protein synthesis using cycloheximide (10 µm, 24 h) in A549 oeNLRP11 (H), SPCA1 shNLRP11 (J), and H1299 sgNLRP11 (L) cell lines and corresponding control cells. M) The expression of NLRP11, vimentin, and vimentin‐K104Ac were detected using IHC in LUAD tissues with different TNM stages.

RNA‐seq from the human protein ATLAS database suggested that the expression of vimentin mRNA in LUAD tissues was approximately seven times lower than that in bronchial epithelial tissues (Figure S4A, Supporting Information); however, IHC from the ATLAS results showed that the positive rate of vimentin protein expression in lung cancer tissue was considerably higher than that in type I and II alveolar cells (Figure S4B, Supporting Information). Here, we identified a novel vimentin, vimentin‐K104Ac, which binds to NLRP11 in H1299 cells (Figure 2A). To explore the effect of this previously unreported modification site on vimentin, we constructed vimentin K104Q (constitutive acetylation) and K104R (deacetylation) plasmids and observed that their expression was higher in 293T cells transfected with vimentin K104Q and K104R plasmids of the same quality than in those transfected with vimentin‐WT plasmids. Simultaneously, vimentin expression was higher in 293T cells transfected with vimentin K104Q than in cells transfected with K104R (**Figure** **3**A). We prepared polyclonal antibodies against vimentin‐K104Ac and dot blotting was used to verify the specificity of the antibody (Figure S4C, Supporting Information). Next, we detected the effects of NLRP11 on vimentin‐K104Ac, and the results showed that NLRP11 could enhance the expression of endogenous vimentin and exogenous vimentin‐WT, instead of exogenous vimentin‐K104Q, indicating that NLRP11‐regulation of vimentin depends on the K104 site. Moreover, NLRP11 markedly increased K104 acetylation of endogenous vimentin and exogenous vimentin‐WT (Figure 3B,C). We further noted that the expressions of vimentin‐K104Ac in LUAD cell lines and early‐stage LUAD tissues was noticeably higher than that in the human bronchial epithelial (HBE) cell lines and their corresponding adjacent normal tissues (Figure S4D,E, Supporting Information). Furthermore, NLRP11 also increased vimentin‐K104Ac protein levels (Figure 3D–F). Non‐histone protein acetylation often involves protein degradation, because it competes for lysine ubiquitination at the same position.^[^ ^34^ ^]^ After inhibition of proteasome activity by ps341, the effects of NLRP11 on vimentin were attenuated (Figure 3G,I,K). Cycloheximide was used to inhibit protein synthesis; therefore, we observed that NLRP11 maintained the stability of vimentin (Figure 3H,J–L). These findings demonstrate that NLRP11 enhances the expression of vimentin‐K104Ac, a more stable form of vimentin, and maintains the stability of the vimentin protein by altering the K104 site. In clinical samples, the NLRP11, vimentin, and vimentin‐K104Ac were detected in LUAD tissues at different tumor node metastasis (TNM) stages using IHC, and the results demonstrated that the expression of NLRP11 and vimentin‐K104Ac in distant metastatic foci from stage IV patients was markedly higher than that in primary foci (Figure 3M; Figure S4F,G, Supporting Information). Vimentin was often highly expressed in the mesenchymal tissue rather than in the tumor, indicating that the expression of NLRP11 and vimentin‐K104Ac was more tumor‐specific (Figure 3M). In addition, the expression of NLRP11 and vimentin‐K104Ac exhibited a strong positive correlation with LUAD tissues (Spearman's R = 0.5733, *P* = 0.0012).

### 2.4 NLRP11 Suppresses K48‐Linked Ubiquitination of Vimentin

**Figure 4:**
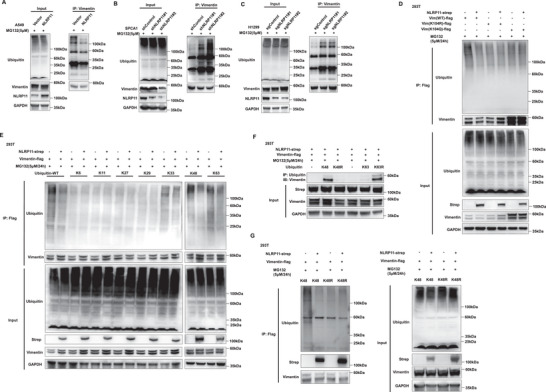
NLRP11 suppresses K48‐linked ubiquitination of vimentin in a manner dependent on K104. A–C) Ubiquitin‐IP was used to detect the ubiquitination of vimentin in the A549 oeNLRP11 (A), SPCA1 shNLRP11 (B), and H1299 sgNLRP11 (C) cell lines and corresponding control cells. D) The effect of NLRP11 on the ubiquitination of vimentin‐WT, K104R, and K104Q was estimated using ubiquitin‐IP. E) The effects of NLRP11 on ubiquitination of WT, K6, K11, K27, K29, K33, K48, and K63 of vimentin were assessed. F) The combinations of vimentin with K48 and K63 ubiquitination were detected using co‐IP. G) The regulation of NLRP11 on ubiquitination‐K48 and K48R of vimentin was tested using ubiquitin‐IP.

To verify whether NLRP11 mediated‐K104 acetylation affects protein stability through vimentin ubiquitination, we used ubiquitin‐IP to detect vimentin ubiquitination in cell lines. The results showed that NLRP11 overexpression markedly attenuated vimentin ubiquitination and that NLRP11 suppression enhanced it (**Figure** **4**A–C). Simultaneously, the ubiquitination of vimentin‐K104Q and K104R was perceptibly lower than that of vimentin‐WT, suggesting that K104 is an important site affecting the ubiquitination of vimentin, whereas NLRP11 could prominently inhibit the ubiquitination of vimentin‐WT and not mutant vimentin at K104 (Figure 4D). Different ubiquitin chain types have different effects on target proteins, including K6, K11, K27, K29, K33, K48, and K63.^[^ ^35^, ^36^ ^]^ Here, we observed that NLRP11 considerably inhibited K48‐linked ubiquitination of vimentin (Figure 4E,G) and interfered with the binding of vimentin and K48‐linked ubiquitin (Figure 4F). Furthermore, LC–MS/MS revealed that the vimentin protein in H1299 cells undergoes ubiquitination at K262, K168, and K188, whereas the K104Q mutation mediates deubiquitination at K168 and K262 (Figure S5A–F, Supporting Information). Collectively, these results indicate that NLRP11 inhibits K48‐linked ubiquitination of vimentin.

### 2.5 NLRP11 Bridges the Binding of KAT7 and Vimentin in the Cytoplasm

**Figure 5:**
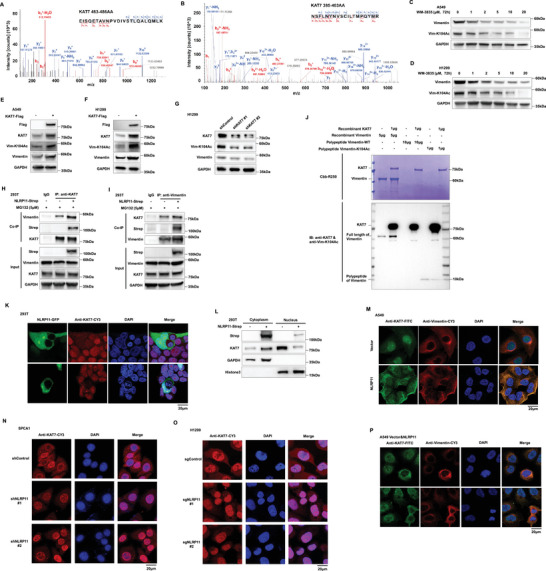
Cytoplasmic KAT7 recruited by NLRP11 directly mediates acetylation of vimentin K104. A) The secondary mass spectrogram shows the peptide fragment of KAT7 binding to the NLRP11 protein in H1299 cell. B) The secondary mass spectrogram shows the peptide fragment of KAT7 binding to the vimentin protein in H1299 cell. C,D) The effects of WM‐3835 (KAT7 inhibitor) on the expression of vimentin and vimentin‐K104Ac were detected using western blot in A549 (C) and H1299 (D) cells. E,F) The expression levels of vimentin and vimentin‐K104Ac were detected in A549 (E) and H1299 (F) cells overexpressing KAT7 using western blotting. G) The expression levels of vimentin and vimentin‐K104Ac were detected in H1299 shKAT7 cells using western blotting. H,I) The combination of vimentin and KAT7 and the effects of NLRP11 on this combination were assessed using co‐IP. J) The effects of recombinant KAT7 on the acetylation of recombinant vimentin, polypeptide vimentin‐WT, and vimentin‐K104Ac were detected using an in vitro assay. K,L) The relationship between the expression of NLRP11 and the subcellular localization of KAT7 was analyzed using immunofluorescence combined with confocal microscopy (K) and western blot (L). M) The effects of NLRP11 on the colocalization of vimentin and KAT7 were estimated using immunofluorescence combined with confocal microscopy in A549 NLRP11 cell lines. N,O) The subcellular localization of KAT7 was analyzed using immunofluorescence combined with confocal microscopy in SPCA1 shNLRP11 and H1299 sgNLRP11 cells. P) The relationships between the subcellular localization of KAT7 and the expression of vimentin in cocultures of A549 vector and NLRP11 cells using immunofluorescence and confocal microscopy.

These results suggest that NLRP11 can mediate the acetylation of vimentin‐K104; however, it has no acetyltransferase function. Therefore, we screened three acetyltransferases using LC‐MS/MS, including KAT7 (**Figure** **5**A), acetyl‐CoA acetyltransferase 1 (ACAT1), and acetyl‐CoA acetyltransferase 2 (ACAT2), which could potentially bind to NLRP11. The peptide segments of KAT7 were also found to interact with vimentin using co‐IP combined with LC–MS/MS (Figure 5B). ACAT1 and ACAT2 may mediate changes in the acetylation of target proteins by affecting the concentration of local acetylation donors, such as Acetyl‐CoA,^[^ ^37^, ^38^, ^39^ ^]^ suggesting that ACAT1 and ACAT2 may affect the acetylation of vimentin without binding to it. Furthermore, WM‐3835 (a KAT7 inhibitor) appreciably inhibited the protein expression of vimentin and vimentin‐K104Ac (Figure 5C,D) but not that of the ACAT1 (K‐604 dihydrochloride) and ACAT2 (avasimibe) inhibitors (Figure S6A,B, Supporting Information). KAT7 overexpression considerably promoted the expression of vimentin and vimentin‐K104Ac (Figure 5E,F), whereas KAT7 suppression inhibited the expression of vimentin and vimentin‐K104Ac (Figure 5G). Molecular docking analysis predicted the combination of vimentin and KAT7 using AutoDock Vina v.1.2.2 software (Figure S5C, Supporting Information), and GPS‐Prot Protein‒Protein Interactions Analysis (http://gpsprot.org/index.php) revealed that vimentin may bind to KAT7 (Figure S6D, Supporting Information). Co‐IP results suggested that there was a close binding between KAT7 and vimentin, which could be strengthened by NLRP11 (Figure 5H,I). An in vitro acetylation assay confirmed that KAT7 directly mediated the acetylation of vimentin at K104 (Figure 5J). Confocal and western blot results indicated that the overexpression of NLRP11 mediated the distribution of KAT7 in the cytoplasm (Figure 5K–M), and the co‐localization signal of KAT7 and vimentin was noticeably enhanced by NLRP11 overexpression (Figure 5M), whereas the suppression of NLRP11 attenuated the distribution of KAT7 in the cytoplasm (Figure 5N,O). Moreover, cytoplasmic localization of KAT7 increased vimentin expression (Figure 5P). Kaplan–Meier plot analysis demonstrated that high KAT7 expression was related to poor prognosis in patients with LUAD (Figure S6E, Supporting Information). Further, the evaluation of its biological effects revealed that WM‐3835 profoundly inhibited LUAD cell proliferation (Figure S6F,G, Supporting Information), invasion, migration (Figure S6H,I, Supporting Information), clonal formation, and tumor sphere formation (Figure S6J,K, Supporting Information). In LUAD cell lines, we observed that high expression of vimentin‐K104Ac was always accompanied by high expression of NLRP11 and KAT7 in H1299, H1993, SPCA1, and A549 cells (Figure S5L, Supporting Information). Moreover, KAT7 overexpression promoted tumor sphere formation, invasion, and migration (Figure S6M–P, Supporting Information) but had no noticeable impact on cell proliferation (Figure S6O,P, Supporting Information), which may be because of the complex target genes of KAT7. In addition, the combination of IP and LC–MS/MS results showed that HDAC2 was most likely the deacetylase of vimentin in LUAD cells (Figure S6Q,R, Supporting Information). Collectively, these results indicate that NLRP11 mediates the cytoplasmic localization of KAT7 and promotes the binding of KAT7 to vimentin.

### 2.6 Both Vimentin‐K104Ac and NLRP11 Accelerate LUAD Progression

**Figure 6:**
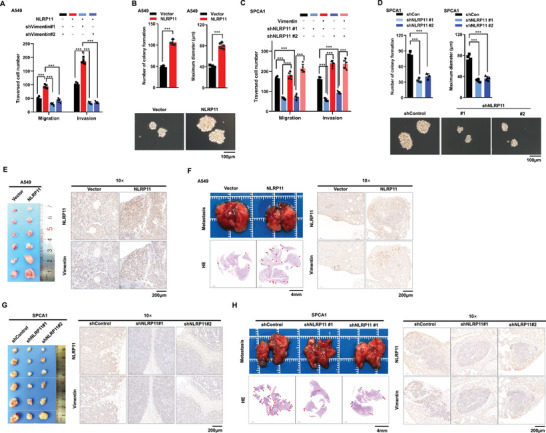
NLRP11 promotes the malignant phenotype of LUAD. A) Quantification and comparison of penetrated cells were measured using Transwell invasion and migration assays in A549 cells overexpressing NLRP11 and vimentin knockdown cells. B) Quantification and comparison of clonalities and sphere‐forming cells were detected using colony formation (left) and tumor sphere formation assays (right) in A549 cells overexpressing NLRP11 and control cells. C) Quantification and comparison of penetrated cells were measured using Transwell invasion and migration assays in SPCA1 shNLRP11 and overexpressing vimentin cells. D) Quantification and comparison of clonalities and sphere‐forming cells were detected using colony formation (left) and tumor sphere formation assays (right) in SPCA1 shNLRP11 and control cells. E) Xenograft tumor assays were used to estimate the proliferation of A549 cells overexpressing NLRP11 and control cells in vivo. F) Experimental metastases were established via tail vein injection to detect the metastatic abilities of A549 cells overexpressing NLRP11 and control cells, and IHC was used to detect the expression of NLRP11 and vimentin in vivo. G) Xenograft tumor assays were used to estimate the proliferation of SPCA1 shNLRP11 and control cells in vivo. H) Experimental metastases were established via tail vein injection to detect the metastatic abilities of SPCA1 shNLRP11 and control cells, and IHC was used to detect the expression of NLRP11 and vimentin in vivo. (** *P* < 0.01, *** *P* < 0.001).

Vimentin, a key marker of EMT, is closely associated with the malignant phenotype of lung cancer.^[^ ^40^, ^41^ ^]^ Kaplan–Meier plot analysis revealed that high vimentin expression was associated with shortened survival time in patients with LUAD (Figure S7A,B, Supporting Information) and that there were higher levels of vimentin‐K104Ac in the serum of patients with early‐stage LUAD than those in healthy individuals (Figure S7C, Supporting Information). This evidence suggests that vimentin‐K104Ac may be an important factor that promotes vimentin stability in LUAD. Therefore, we overexpressed vimentin and vimentin‐K104Ac in the A549 and H1299 cell lines (Figure S7D,E, Supporting Information) and found that the upregulation of vimentin and vimentin‐K104Ac facilitated invasion, migration (Figure S7F,G, Supporting Information), clonal formation, and tumor sphere formation (Figure S7H,I, Supporting Information). Moreover, vimentin‐K104Ac promoted malignant behavior to a greater extent compared to vimentin alone (Figure S7D–I, Supporting Information). Based on the effect of NLRP11 on vimentin‐K104Ac, we evaluated the biological effects of NLRP11 on LUAD cells in vitro and in vivo. The results revealed that the overexpression of NLRP11 markedly promoted metastasis (**Figure** **6**A) (Figure S8A, Supporting Information), proliferation (Figure S8B, Supporting Information), clonality (Figure 6B) (Figure S8C, Supporting Information), and stem cell characteristics (Figure 6B) of A549 cells. Knockdown or knockout of NLRP11 noticeably inhibited the malignant phenotype of SPCA1 and H1299 cells (Figure 6C,D) (Figure S8D–I, Supporting Information) in vitro. In in vivo, the overexpression of NLRP11 increased the growth of xenograft tumors (Figure 6E) and circulatory metastasis (Figure 6F) and enhanced the expression of vimentin (Figure S9A,B, Supporting Information), whereas knockdown or knockout of NLRP11 suppressed the growth and metastasis of SPCA1 and H1299 cells (Figure 6G,H) and attenuated the expression of vimentin (Figure 6G,H) (Figure S9C–F, Supporting Information). However, in PC9 cells, which hardly express vimentin, NLRP11 overexpression had no significant effect on the malignant behavior of the cells (Figure S10A–C, Supporting Information). Furthermore, WM‐3835 suppressed the increase in vimentin, vimentin‐K104Ac, and metastasis mediated by NLRP11 in A549 cells (Figure S10D,E, Supporting Information). Briefly, these results demonstrate that NLRP11 promotes the malignant phenotype of LUAD cells via KAT7/vimentin.

### 2.7 NOD‐Like Inhibitor MCC950 Suppresses the Malignant Behavior of LUAD Cells with High Expression of Vimentin

**Figure 7:**
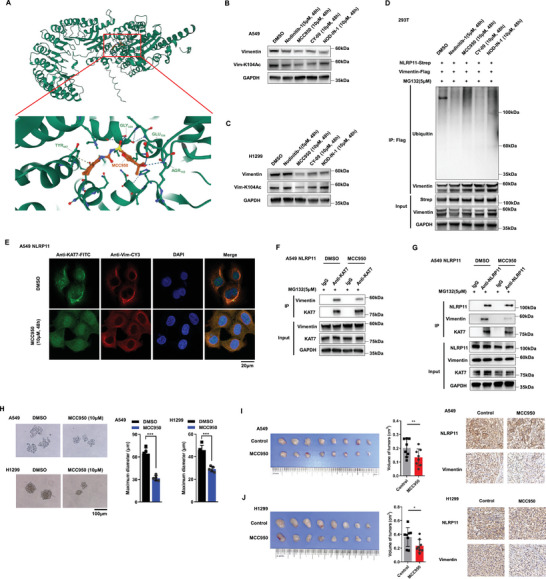
MCC950 constricts the malignant behavior of LUAD cells with high expression of vimentin. A) The binding sites of MCC950 and NLRP11 protein were simulated using AutoDock Vina v.1.2.2 molecular docking analysis, whose low binding energy is −8.109 kcal mol^−1^. B,C) The effects of DMSO, Nodinitib‐1 (5 µm), MCC950 (10 µm), CY‐09 (10 µm), and NOD‐IN‐1 (10 µm) on the expression of vimentin and vimentin‐K104Ac were detected using western blot in A549 and H1299 cells. D) Ubiquitin‐IP was used to measure the effects of DMSO, Nodinitib‐1 (5 µm), MCC950 (10 µm), CY‐09 (10 µm), and NOD‐IN‐1 (10 µm) on the ubiquitination of vimentin. E) Confocal microscopy was performed to test the impacts of MCC950 on the colocalization of KAT7 and vimentin in A549 cells overexpressing NLRP11. F) Co‐IP was performed to explain the influence of MCC950 on the combination of KAT7 and vimentin in A549 cells overexpressing NLRP11. G) Co‐IP was performed to detect the influence of MCC950 on the combination of NLRP11 and KAT7 or vimentin in A549 cells overexpressing NLRP11. H) Tumor sphere formation assays were used to estimate the sphere‐forming capability of A549 and H1299 cells treated with MCC950. I,J) Xenograft tumor assays were used to estimate the proliferation of A549 (I) and H1299 (J) cells treated with MCC950, and IHC was used to detect the expression of NLRP11 and vimentin in vivo (* *P* < 0.05, ** *P* < 0.01, *** *P* < 0.001).

The above‐mentioned evidence indicates that inhibiting NLRP11 may be an effective way to prevent lung cancer. NOD, also known as NACHT, is a common domain of the NLR family, whose members function as molecular switches with an ADP‐bound inactive state and an ATP‐bound active state.^[^ ^42^, ^43^ ^]^ NOD‐like inhibitors always affect the function of NLRs by combining with the NOD domain. Therefore, to evaluate the affinity of the candidate NOD‐like inhibitors to NLRP11, we performed molecular docking analysis with AutoDock Vina v.1.2.2. Four drug candidates (Nodinitib‐1, MCC950, CY‐09, and NOD‐IN‐1) were obtained from 18 NOD‐like inhibitors, which had low binding energies of −6.703, −8.109, −7.094, and −6.810 kcal mol^−1^, respectively, and all were potentially bound to NLRP11 at Tyr^547^ (**Figure** **7**A) (Figure S11A–C, Supporting Information). We found that 10 µm MCC950 considerably suppressed vimentin and vimentin‐K104Ac protein expression in A549 and H1299 cells (Figure 7B,C). In addition, MCC950 facilitated the ubiquitination of vimentin (Figure 7D) and attenuated the colocalization (Figure 7E) and binding capability (Figure 7F) of KAT7 and vimentin. Furthermore, we predicted the binding model between NLRP11 and KAT7 or vimentin using AutoDock Vina v.1.2.2 software (Figure S11D,E, Supporting Information), and the results showed that the possible binding spatial structure of NLRP11 and vimentin was similar to the predicted position of MCC950 combined with NLRP11 (Figure S11D, Supporting Information). Co‐IP assays demonstrated that MCC950 interfered with NLRP11 binding to vimentin instead of KAT7 (Figure 7G). Therefore, we evaluated the bioeffects of MCC950 on A549 and H1299 cells, and the results demonstrated that MCC950 markedly inhibited cell proliferation (Figure S11F,G), colony formation (Figure S11H,I, Supporting Information), metastasis (Figure S11J–M, Supporting Information), sphere formation (Figure 7H), and growth of xenograft tumors (Figure 7I,J). These results suggest that MCC950, a small molecule inhibitor of NLRP11, has potential value in restricting vimentin‐positive LUAD cells.

## 3 Discussion

**Figure 8:**
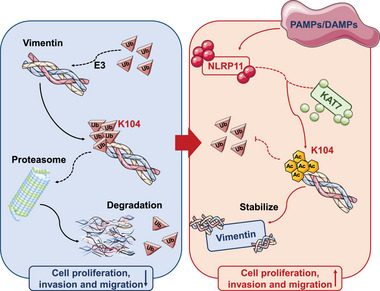
The proposed working model. Under normal conditions, vimentin K104 can bind to ubiquitin and enter the proteasome pathway for degradation. In cancer cells persistently expressing NLRP11, KAT7 tends to translocate from the nucleus to the cytoplasm and is recruited to bind with vimentin by NLRP11, resulting in vimentin acetylation at K104 and inhibition of ubiquitin‐independent proteasomal degradation, thereby contributing to the malignant phenotype related to epithelial–mesenchymal transition in LUAD cells.

To date, there is no consensus on the main drivers of early‐stage LUAD. Identification of carcinogenesis induced by inflammation is pivotal not only for revealing mechanistic insights into cancer but also for developing novel cancer therapy strategies. In this study, we identified an inflammation‐related molecule, NLRP11, as a recruiter that connects KAT7 with vimentin, thereby mediating the acceleration of EMT in partial LUAD cells (**Figure** **8**).

First, the abnormal state of cytokines, which is the most prominent molecular feature of early LUAD, was observed at the transcriptional level. As direct regulators of cytokines, NLRs play a connecting role in the cellular response to stress stimulation and participate in the malignant transformation and evolution of tumor cells.^[^ ^6^ ^]^ We observed that NLRP11 was the most significantly differentially expressed gene among NLRs in early‐stage LUAD and adjacent tissues, and its high expression predicted a poor prognosis, suggesting that NLRP11 may be involved in the occurrence and development of LUAD. Previous studies have indicated that NLRP11 regulates inflammasome activation in human macrophages and lymphocytes and its mechanisms include the following: NLRP11 suppresses NLRP3‐mediated caspase‐1 activation by binding to DDX3X and reducing its phosphorylation,^[^ ^33^ ^]^ but NLRP11 also interacts with NLRP3 and ASC and promotes NLRP3 inflammasome activation by preventing inflammasome assembly, NLRP3 and ASC polymerization, caspase‐1 activation, pyroptosis, and cytokine release,^[^ ^32^ ^]^ which reflects the controversial role of NLRP11 in the inflammatory response. In addition, NLRP11 can degrade TRAF6 by recruiting the ubiquitin ligase, RNF19A, to catalyze K48‐linked ubiquitination at multiple sites and restrain the production of type I IFNs and virus‐induced apoptosis in the mitochondria.^[^ ^31^, ^44^, ^45^ ^]^ These reports indicate that NLRP11 functions by interacting with proteins.

When researching the underlying mechanism, we found that NLRP11 directly binds to vimentin, and an unreported acetylation modification of vimentin at K104 was observed. The functional mechanisms of non‐histone protein acetylation involve the regulation of enzymes, protein degradation, protein–protein interactions, and subcellular localization.^[^ ^34^ ^]^ Acetylation of vimentin at K120 has been reported to promote the EMT and migration ability of hepatocellular carcinoma,^[^ ^28^ ^]^ but its mechanism has not been fully elucidated. The results of this study showed that, compared to vimentin‐WT, the expression of exogenous vimentin‐K104Q and vimentin‐K104R was more stable in 293T cells. Simultaneously, NLRP11 mediates a noticeable increase in vimentin and vimentin‐K104Ac protein expression, and this regulation depends on the K104 site. Competitive binding of the same lysine with ubiquitination is one of the central mechanisms of acetylation affecting protein stability, which affects the degradation of the ubiquitin proteasome pathway.^[^ ^46^, ^47^, ^48^ ^]^ Notably, if K104 de‐ubiquitination mediated by the K104 mutation is the only factor leading to vimentin accumulation, the expression of vimentin in cells transfected with K104Q and K104R should be the same. However, the effect of K104Q on vimentin accumulation was stronger than that of K104R, suggesting that K104Ac may be involved in vimentin accumulation via crosstalk with other sites. LC–MS/MS revealed that the K104Q mutation can mediate de‐ubiquitination of vimentin at K168 and K262 in H1299 cells, indicating that there may be mutual crosstalk between the K104 and K168/K262 sites, which synergistically inhibit the ubiquitination degradation of vimentin. Therefore, we considered that the stability of vimentin‐K104Ac may be related to a reduction in ubiquitination. In subsequent experiments, we found that NLRP11 can inhibit the K48‐ubiquitination of vimentin and degradation of the ubiquitin‐proteasome pathway, and this inhibition is also dependent on the K104 site. To clarify the functional domain of NLRP11, we found that the expression of vimentin could be promoted only when NLRP11 was complete and that both the PYD and LRR domains could be combined with vimentin, indicating that NLRP11 structural integrity is necessary for regulating vimentin expression. In the analysis of clinical samples in this study, NLRP11 and vimentin‐K104Ac were highly expressed in early‐stage LUAD tissues and were enhanced in distant metastatic foci, indicating important roles of NLRP11 and vimentin‐K104Ac in the progression of LUAD. Compared to vimentin, NLRP11 and vimentin‐K104Ac showed better tumor specificity, reflecting the differences between EMT‐mediated tumor cells and mesenchymal cells. Although not all occurrences and metastases of LUAD are related to the positive expression of vimentin, the expression of NLRP11 is often accompanied by the expression of vimentin and vimentin‐K104Ac in LUAD cells, which indicates that LUAD still has a trace of similarity in dramatic individual differences.

However, because NLRP11 lacks acetylation‐catalytic ability, we further investigated the mass spectrometry results and found that the acetyltransferase, KAT7, potentially bound to NLRP11, may be involved in mediating the acetylation of vimentin. Recent reports have demonstrated that cytoplasmic KAT7 is involved in the development of malignant tumors depending on its acetyltransferase activity.^[^ ^49^ ^]^ In this study, co‐IP and confocal microscopy confirmed that cytoplasmic KAT7 significantly bound to vimentin. NLRP11 significantly enhanced the binding strength of KAT7 and vimentin, which was mediated by NLRP11 promoting KAT7 translocation from the nucleus to the cytoplasm. We used WM‐3835 to specifically inhibit KAT7 and found that the increased acetylation of vimentin, mediated by NLRP11, was inhibited. Therefore, we believe that NLRP11‐mediated acetylation of vimentin at K104 depends on cytoplasmic KAT7 levels.

Since the NLRP11/KAT7/vimentin‐K104Ac pathway is a major factor in vimentin stability, we first measured the bioeffects of NLRP11 in vimentin‐positive (+) and vimentin‐negative (−) LUAD cells. NLRP11 enhanced the expression of vimentin protein in vimentin‐positive LUAD cells but could not induce vimentin expression in cells that barely expressed vimentin, indicating that the expression of vimentin protein is a prerequisite for NLRP11 to play a role in this pathway and that NLRP11 regulates the expression of vimentin at the post‐transcriptional level. The unique regulatory mechanism of NLRP11 is reflected in its biological effects. Overexpression of NLRP11 appreciably promoted the malignant phenotype of vimentin‐positive LUAD cells in vivo and in vitro, whereas the knockdown or knockout inhibited the malignant phenotype; however, these effects were not significantly different in vimentin‐negative LUAD cells. As an acetyltransferase directly involved in this pathway, KAT7 participates in the malignant progression of LUAD. WM‐3835 noticeably inhibits the malignant phenotype of vimentin‐positive LUAD cells. Although overexpression of KAT7 had no significant effect on cell proliferation, this may be caused by the complexity of KAT7 targets. Moreover, vimentin‐K104Ac has a stronger ability to promote malignancy compared to vimentin‐WT, which is related to the stability of vimentin‐K104Ac. MCC950, selected from 18 candidates, had a low binding energy of −8.109 kcal mol^−1^, indicating highly stable binding. It considerably reduced the expression levels of vimentin and vimentin‐K104Ac and inhibited the malignant phenotype of cells. In an in vivo model of salt‐sensitive hypertension, it should be noted that MCC950 attenuated the expression of vimentin and several inflammatory/injury markers in the kidney.^[^ ^50^ ^]^ Mechanistically, MCC950 can also impair the combination of NLRP11 and vimentin and inhibit the acetylation of vimentin mediated by KAT7 at K104, suggesting that MCC950 has potential antitumor value in patients with vimentin‐K104Ac‐positive lung cancer.

In summary, we first revealed a novel acetylation of vimentin at K104 in LUAD cells, which was mediated by KAT7 recruited by NLRP11. Acetylation of vimentin at K104 noticeably inhibited the degradation of the ubiquitin‒proteasome pathway, which is a pivotal factor in stabilizing vimentin and an important supporting factor for maintaining the vimentin‐related malignant phenotype. The NLRP11/KAT7/vimentin‐K104Ac pathway may be a crucial in the inflammation‐mediated EMT in tumor cells. Our findings will promote the development of new molecular classifications for LUAD, which will further optimize the diagnosis, evaluation, and individualized treatment of LUAD.

## 4 Experimental Section

### Cell Lines and Culture

Human LUAD cells A549, PC9, H358, H1299, H1993, HCC827, SPCA1, H1975, and HBE cells from American Type Culture Collection were cultured in RPMI 1640 or DMEM/F12 medium (Gibco, CA, USA) supplemented with 10% fetal bovine serum (Gibco) and penicillin/streptomycin at 37 °C in a humidified atmosphere with 5% CO_2_. All cells were authenticated using mycoplasma detection and DNA fingerprinting and were subcultured 1:2 to 1:3 when they reached 70%–90% density.

### Patients and Tissue Samples

In this study, the inclusion criteria for early LUAD were as follows: the tumor tissue was consistent with the ground glass opacity diagnosis on computed tomography imaging, single nodule diameter was <1.0 cm; LUAD was diagnosed via pathology, and the AJCC/TNM stage was classified as IA. 1) RNA‐seq was performed on 23 early LUAD tissues and their corresponding adjacent noncancerous tissues from November 2018 to April 2019. 2) TMT‐quantitative proteomics was performed on six early stage LUAD tissues and their corresponding adjacent noncancerous tissues. All tissues were collected after obtaining informed consent from patients at Xiangya Hospital Central South University. The study protocol was approved by the Institutional Ethics Review Board of the Xiangya Hospital Central South University. All samples were snap‐frozen and stored in liquid nitrogen.

### RNA Sequencing

BGI Genomics Co., Ltd. (https://www.genomics.cn/) was used for RNA sequencing of tissue samples in this study. Total RNA was isolated from tissues using TRIzol reagent (Thermo Fisher Scientific, MA, USA) and quantified using a NanoDrop and Agilent 2100 bioanalyzer (Thermo Fisher Scientific). An mRNA library was constructed and amplified with phi29 to generate DNA nanoballs (DNBs) containing more than 300 copies of one molecule. DNBs were loaded into the patterned nanoarray and paired‐end 100‐base reads were generated on the BGIseq500 platform (BGI‐Shenzhen, China).

### Plasmids and Cell Treatment

Plasmids contained GV367‐NLRP11, GV248‐shNLRP11#1‐3, GV358‐Vimentin‐3Flag, GV358‐KAT7‐3Flag, GV248‐shVimentin#1‐3, and their corresponding control vectors (GeneChem, Shanghai, China), Plvx‐PYD‐Flag, Plvx‐NOD‐Flag, Plvx‐LRR‐Flag, and their corresponding control vectors (Tsingke, Hunan, China), pSpCas9(BB)−2A‐GFP (Px458), Ubiquitin‐WT, K6, K11, K27, K29, K33, K48, K63, K48R, and K63R (Addgene, MA, USA), and Piggybac transposon vector (PB510B, System Biosciences, CA, USA). PB510B‐NLRP11‐twin‐strep, Px458‐NLRP11‐sgRNA1#, and Px458‐NLRP11‐sgRNA2# were established using molecular cloning. The sequences of sgRNAs targeting NLRP11 were designed by e‐crisp (http://www.e‐crisp.org/E‐CRISP/), and the sequence is as follows: NLRP11‐sgRNA1#: forward: 5′‐CACCGGCTTGGCTGAGCTAATCGCCA‐3′; reverse: 5′‐AAACTGGCGATTAGCTCAGCCAAGCC‐3′. NLRP11‐sgRNA2# forward: 5′‐CACCGGATTCTCTAGATACCACAGC‐3′; reverse: 5′‐AAACGCTGTGGTATCTAGAGAATCC‐3′. Plasmid transfection using Lipofectamine 2000 (Thermo Fisher Scientific) was performed in accordance with the manufacturer's instructions, and puromycin (2 µg ml^−1^) was used to screen positive colonies with stable expression. Inhibition of protein synthesis using cycloheximide was used to determine the rate of protein degradation in the cells. Bortezomib (Ps341) and MG‐132 were used to suppress proteasomal proteolytic activity. The NOD‐like inhibitors, CY‐09, MCC950, Nodinitib‐1, and NOD‐IN‐1 (MedChemExpress, NJ, USA) were used to treat the cells.

### RT‐qPCR

Total RNA was extracted using RNAiso Plus (Takara, Kusatsu, Japan) and cDNA was reverse‐transcribed using the PrimeScript RT reagent kit (Takara). SYBR Green I Master Mix was used for qPCR. The primer sequences used for performing RT‐qPCR are as follows: NLRP11 forward, 5′‐ AGAGGTAGATTGCTGCACGA‐3′; NLRP11 reverse, 5′‐ ACAGTACACGTGATCCAGCA‐3′. NLRP7 forward, 5′‐ CTTTGCAGGAAACACAGGCT‐3′; NLRP7 reverse, 5′‐ CCATGGGGTCTTCTGTAGCA‐3′. NLRP4 forward, 5′‐GACTTGATGGAGAAACGGCC‐3′; NLRP4 reverse, 5′‐ CTTCCATGGCTCTTTTCGGG‐3′. Vimentin forward, 5′‐CAGGAGGCAGAAGAATGGTACAAA‐3′; Vimentin reverse, 5′‐GGCGTTCCAGGGACTCATTG‐3′. β‐actin forward, 5′‐CACCATTGGCAATGAGCGGTTC‐3′; β‐actin reverse, 5′‐AGGTCTTTGCGGATGTCCACGT‐3′. The expression of β‐actin was used as an internal control, and the relative expression of mRNA was calculated using the standard 2^−ΔΔ^Ct method.

### Western Blot Analysis

Total protein was isolated using IP lysis buffer (Beyotime, Shanghai, China) with 1× Complete Protease Inhibitor Cocktail (Sigma‒Aldrich, MO, USA). Protein supernatants were added to 1× loading buffer, boiled for 5 min, and subjected to 10% SDS‐polyacrylamide gel electrophoresis (SDS‐PAGE). Subsequently, the protein bands were transferred to polyvinylidene fluoride membranes (Millipore, MA, USA) for 2–3 h at a constant current of 290 mA. 5% skim milk was used to block the membranes for 2 h. The protein bands were incubated with primary antibodies against NLRP11 rabbit pAb (1:1000 dilution) (A12132, Abclonal, Wuhan, China), vimentin rabbit pAb (1:5000 dilution) (10366‐1‐AP, Proteintech, IL, USA), vimentin mouse mAb (1:50 000 dilution) (60330‐1‐Ig, Proteintech, USA), GAPDH rabbit pAb (1:5000 dilution) (10494‐1‐AP, Proteintech, USA), ubiquitin rabbit pAb (1:500 dilution) (10201‐2‐AP, Proteintech, USA), KAT7 rabbit pAb (1:1000 dilution) (13751‐1‐AP, Proteintech, USA), ubiquitin mouse mAb (1:1000 dilution) (#3936, Cell Signaling Technology, MA, USA), and vimentin‐K104Ac (1:1000 dilution) (PTMBio, Hangzhou, China) for 12–14 h at 4 °C and then incubated with secondary antibodies (1:3000 dilution) (Proteintech, USA) for 1 h. Finally, the bands were detected using chemiluminescence.

### ELISA

Blood solidified naturally at room temperature. The samples were centrifuged at 1000 × *g* for 20 min at 4 °C, and the supernatant was collected as serum from patients with early‐stage LUAD and the Blood Transfusion Department. ELISA coating buffer was mixed with the serum at a ratio of 2:1, and 100 µL well^−1^ was added to a 96‐well plate (Corning Costar 9018) at 37 °C for 1.5 h. Vimentin‐K104Ac polypeptide (0.1 µg) was used as a positive control, and the washing solution was added at intervals of 3 min. This process was repeated four times. The mixture was used to block at room temperature for 1.5 h. The wells were washed for 3 min each time and this process was repeated four times. Then, 1 µg mL^−1^ unconjugated antibodies, including anti‐NLRP11, anti‐vimentin and anti‐vimentin‐K104Ac, and rabbit IgG as a negative control were added and incubated at 37 °C for 1 h. The wells were washed, and HRP‐conjugated antibodies were added and incubated at 37 °C for 1 h. The wells were washed in the same way as described above. TMB chromogen solution (90 L well^−1^) was added to each reaction well, and the washing solution was removed at intervals of 3 min. This process was repeated four times. Then, 50 µL well^−1^ stop solution (2 m H_2_SO_4_) was added to each reaction well, and the optical density (OD) value was immediately determined using a microplate reader at 450 nm.

### In Vitro Acetylation Assays

For in vitro acetylation reaction, 1 µg recombinant KAT7 were incubated with 5 µg recombinant vimentin, 10 µg polypeptide of vimentin‐WT (NTRTNEKVELQELC) or 1 µg polypeptide of vimentin‐K104Ac (NTRTNE‐Acetyl‐K‐VELQELC) in the HAT buffer containing 2 mm acetyl‐coenzyme A (10101893001, Roche, Switzerland), 50 mm pH 8.0 Tris–HCl (ST780, Beyotime, Shanghai, China), 50 mm NaCl, 4 mm MgCl2, 0.1 mM EDTA (C0196, Beyotime, Shanghai, China), 1 mm DTT (ST040, Beyotime, Shanghai, China), and 10% glycerol (ST1348, Beyotime, Shanghai, China) at 37 °C for 1 h. Loading buffer (2×) was added in the above reaction system, and acetylation of vimentin was detected using western blot with an anti‐vimentin‐K104Ac antibody.

### LC‒MS/MS

OE Biotech Co., Ltd. was responsible for LC‒MS/MS, and the detection process was described previous reports.^[^ ^51^ ^]^

### Cell Proliferation, Colony Formation, Cell Migration and Invasion, Tumor Sphere Formation, Co‐IP, IHC, and Immunofluorescence Assays

These assays were performed as previously reported.^[^ ^52^, ^53^, ^54^, ^55^ ^]^

### Statistical Analysis

For the knockout or knockdown experiments, at least two independent sgRNAs or shRNAs were used to generate the respective cell lines. For overexpression experiments, lentiviruses containing the target gene were prepared multiple times to verify the overexpression efficiency in cells. Cell culture experiments were performed with at least three independent replicates. Except for the animal experiments, all assays were performed with at least three independent replicates. Data were presented as mean ± standard deviation (SD) calculated from *n* = 3. The gene set enrichment analysis is shown in Figures S1A and S2A (Supporting Information) were subjected to KEGG enrichment analysis (Figure S2D, Supporting Information was performed using GO enrichment analyses, and statistical significance (P‐values) was calculated using two‐sided unpaired Student's t‐tests and two‐way analysis of variance (ANOVA) using GraphPad Prism 8.0 (GraphPad Software, Inc.)). The following forms represent different statistical results: NS, non‐significant (*P* > 0.05); * *P* < 0.05; ** *P* < 0.01; and *** *P* < 0.001.

### Ethics Approval Statement

The project was approved by the ethics committee of the Second Xiangya Hospital, Central South University.

## Conflict of Interest

The authors declare no conflict of interest.

## Supporting information

Supporting Information

Supporting Information

## Data Availability

The data that support the findings of this study are available from the corresponding author upon reasonable request.
