# Supplementary material for: The NLRP11 Protein Bridges the Histone Lysine Acetyltransferase KAT7 to Acetylate Vimentin in the Early Stage of Lung Adenocarcinoma
Source: Adv Sci (Weinh). 2023 Jul 9;10(25):2300971. doi: 10.1002/advs.202300971 (PMC10477884; doi:10.1002/advs.202300971)
Supplement: Supplementary file 1 — Supporting Information [file ADVS-10-2300971-s001.pdf]

ADVANCED SCIENCE
Open Access

Supporting Information

for *Adv. Sci.*, DOI 10.1002/advs.202300971

The NLRP11 Protein Bridges the Histone Lysine Acetyltransferase KAT7 to Acetylate Vimentin in the Early Stage of Lung Adenocarcinoma

Rui Yang, Weilin Peng, Shuai Shi, Xiong Peng, Qidong Cai, Zhenyu Zhao, Boxue He, Guangxu Tu, Wei Yin, Yichuan Chen, Yuqian Zhang, Fang Liu, Xiang Wang, Desheng Xiao and Yongguang Tao**

The NLRP11 protein bridges the histone lysine acetyltransferase KAT7 to acetylate vimentin in the early stage of lung adenocarcinoma

Rui Yang, Weilin Peng, Shuai Shi, Xiong Peng, Qidong Cai, Zhenyu Zhao, Boxue He, Guangxu Tu, Wei Yin, Yichuan Chen, Yuqian Zhang, Liu Fang, Xiang Wang, Desheng Xiao* and Yongguang Tao*

* To whom correspondence should be addressed to Desheng Xiao and Yongguang Tao

Email: xdsh96@csu.edu.cn; taoyong@csu.edu.cn

Supplementary figures

Supplemental Figure 1

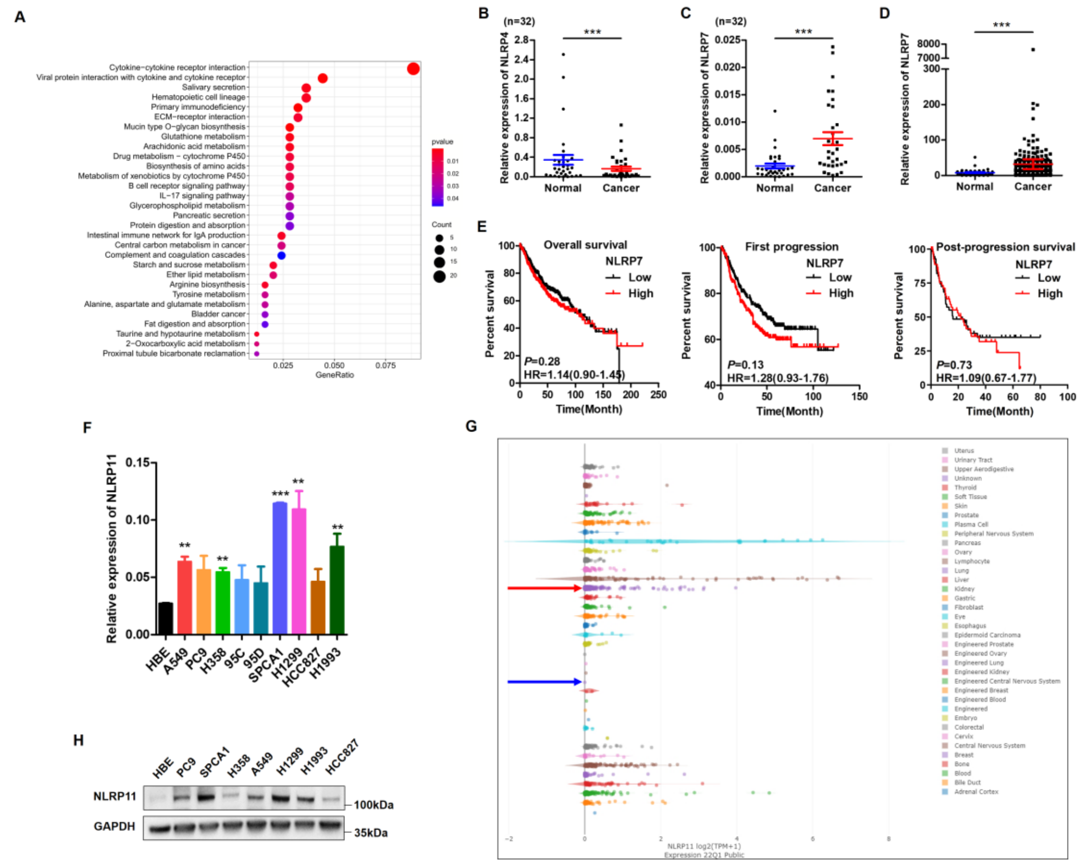

Figure S1. NLRPs are expressed differentially in LUAD. A. The top 30 differential pathways were detected using RNA-seq in early-stage LUAD tissues and their corresponding adjacent normal tissues from 23 patients. Gene Ratio refers to the proportion of differential genes in pathway genes, and the color of bubbles from blue to red indicates the P value from small to large. **B** and **C**. RT–qPCR was used to detect NLRP4 and NLRP7 mRNA in 32 LUAD tissues and their corresponding adjacent normal tissues. **D**. Comparison of NLRP7 mRNA in 59 normal lung tissues and 535 LUAD tissues from the TCGA database. **E**. The relationships between NLRP7 mRNA expression and the prognosis of LUAD, including overall survival (OS), first progression (FP), and post-progression survival (PPS), were analyzed in gene chips from the Kaplan–Meier plotter database. **F**. The expression of NLRP11 mRNA was detected using RT–qPCR in normal bronchial epithelium and LUAD cell lines. **G**. Analysis of NLRP11 mRNA expression in different normal and cancer cell lines from the CCLE database. **H**. Western blotting was used to detect the expression of NLRP11 protein in normal bronchial epithelium and LUAD cell lines (** $P < 0.01$, *** $P < 0.001$).

Supplemental Figure 2

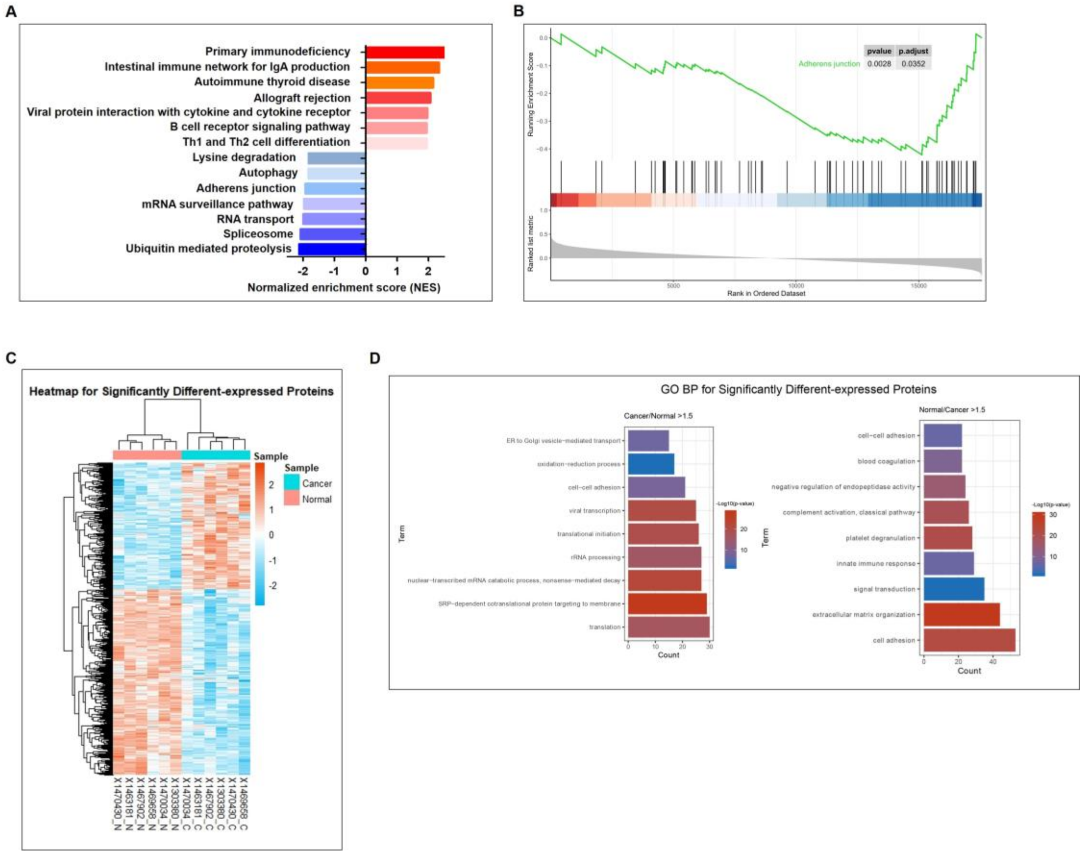

Figure S2. Cell adhesion is involved in the expression of NLRP11 and progression of LUAD. A. GSEA enrichment analysis demonstrated pathways that are positively and negatively related to the expression of NLRP11 mRNA. **B**. The enrichment score of the adherens junction pathway in the genes that are significantly related to NLRP11 mRNA. **C** and **D.** The heatmap and GO analysis of quantitative proteomics exhibited differential protein expression and pathways in six early LUAD and corresponding adjacent noncancerous tissues.

Supplemental Figure 3

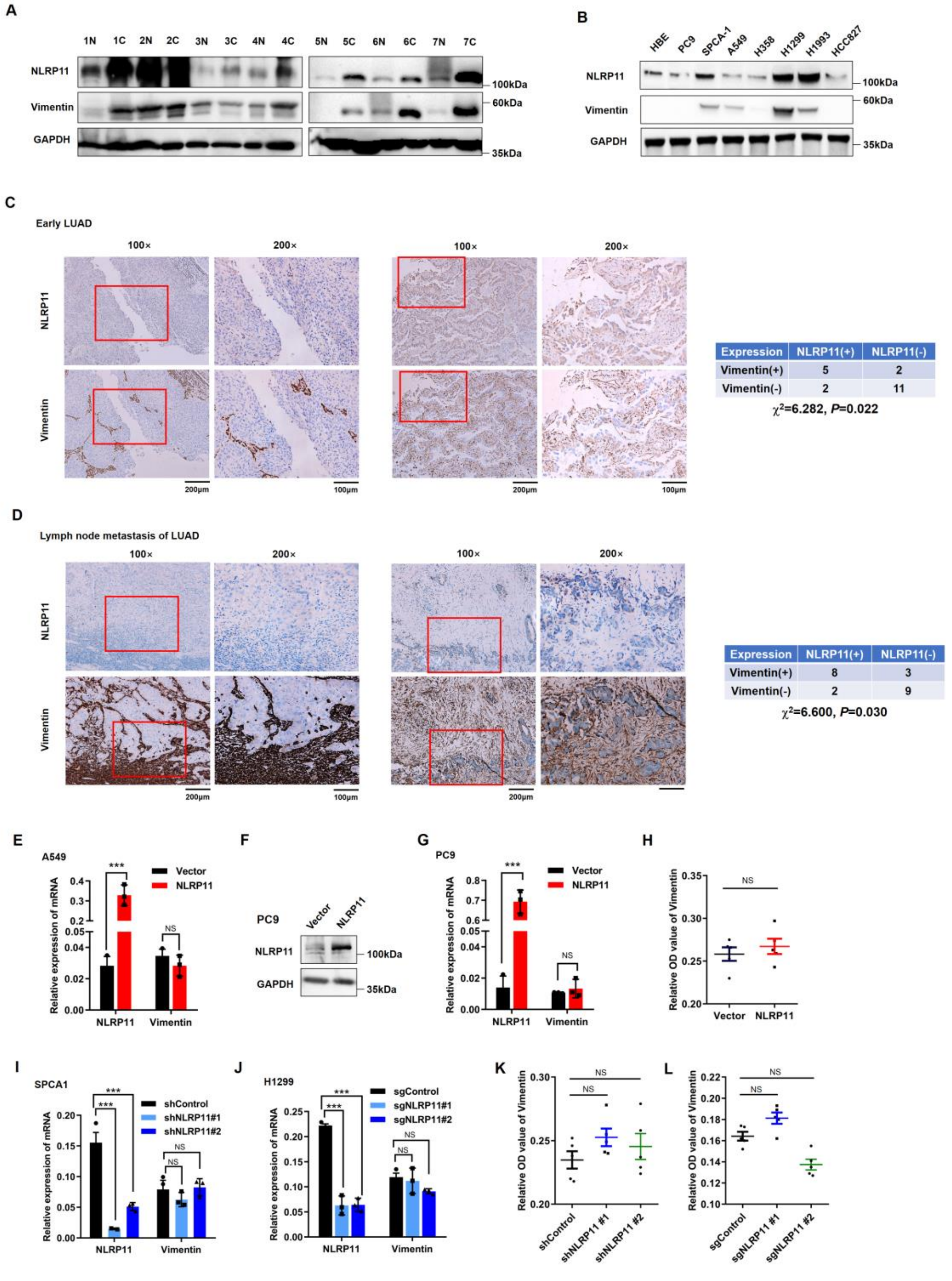

Figure S3. NLRP11 is closely related to vimentin in LUAD. A and **B**. The protein expression levels of NLRP11 and vimentin in seven early-stage LUAD tissues and their corresponding adjacent noncancerous tissues (**A**) and normal bronchial epithelium and LUAD cell lines (**B**) were detected using western blot. **C** and **D**. IHC was used to detect NLRP11 and vimentin in early-stage LUAD (**C**) and lymph node metastasis of LUAD tissues (**D**). **E**. RT–qPCR was used to measure the expression of NLRP11 and vimentin mRNA in A549 cells overexpressing NLRP11. **F** and **G**. western blot (**F**) and RT–qPCR (**G**) were used to detect the expression of NLRP11 protein and mRNA in PC9 vector and NLRP11 cells. **H**. ELISA was used to detect the levels of vimentin in the supernatant of cell culture from A549 overexpressing NLRP11 cell lines. **I** and **J**. RT–qPCR was used to measure the expression of NLRP11 and vimentin mRNA in SPCA1 shNLRP11 (**I**) and H1299 sgNLRP11 (**J**) cell lines. **K** and **L**. ELISA was used to detect the levels of vimentin in the supernatant of cell culture from SPCA1 shNLRP11 (**K**) and H1299 sgNLRP11 (**L**) cell lines (NS, nonsignificant, *** $P < 0.001$).

Supplemental Figure 4

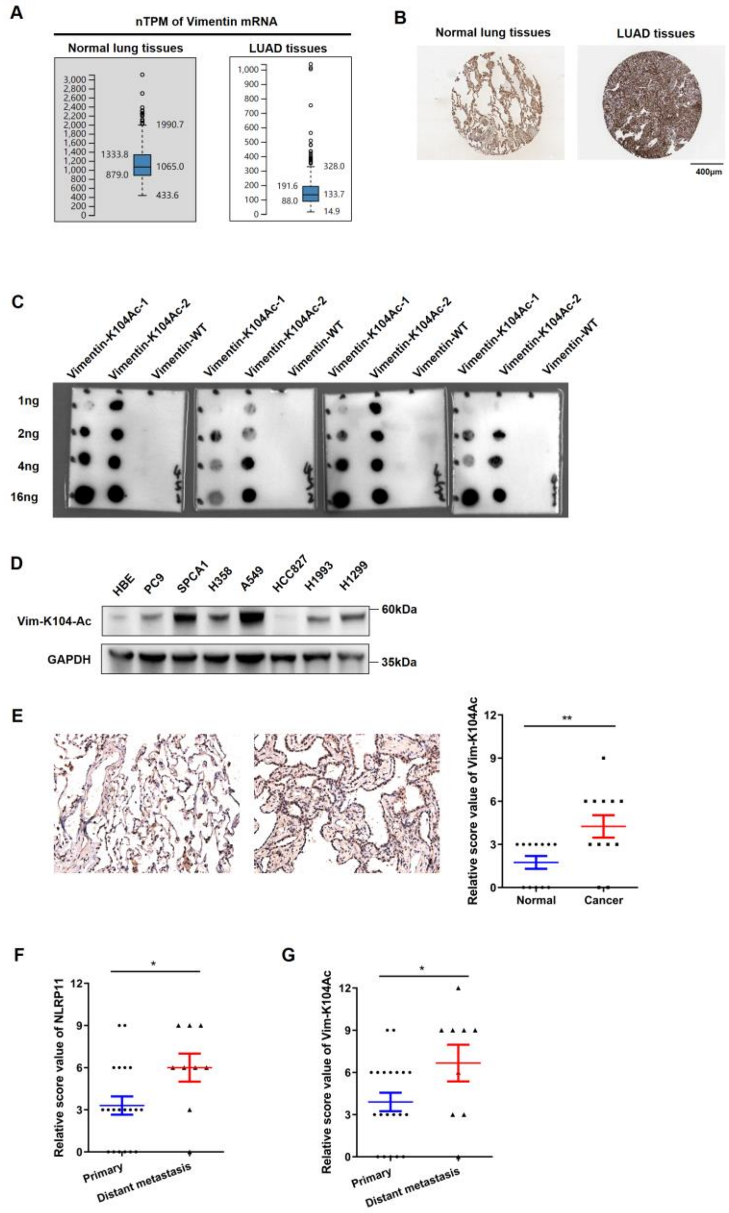

Figure S4. Vimentin and vimentin-K104Ac are highly expressed in LUAD. A. The normalized transcripts per million (nTPM) of vimentin mRNA in normal lung and LUAD tissues from The Human Protein Atlas database were analyzed. **B**. The expression of vimentin protein in alveolar cells and LUAD tissues from The Human Protein Atlas database was analyzed. Vimentin protein was not detected in alveolar cells, and 10 of 11 patients were positive for vimentin protein expression. **C**. Recognition abilities of four anti-vimentin-K104Ac antibodies to vimentin-K104Ac and vimentin-WT polypeptides were measured using dot blot analysis. **D**. The distinctions in vimentin-K104Ac protein expression among lung epithelial cell HBE and LUAD cell lines were detected using western blotting. **E**. IHC were used to measure the expressions of vimentin-K104Ac in 12 pairs of early LUAD and corresponding adjacent normal tissues. **F** and **G**. Comparisons of relative score value of NLRP11 and vimentin-K104Ac in primary focus and distant metastasis foci of LUAD (* $P < 0.05$, *** $P < 0.001$).

Supplemental Figure 5

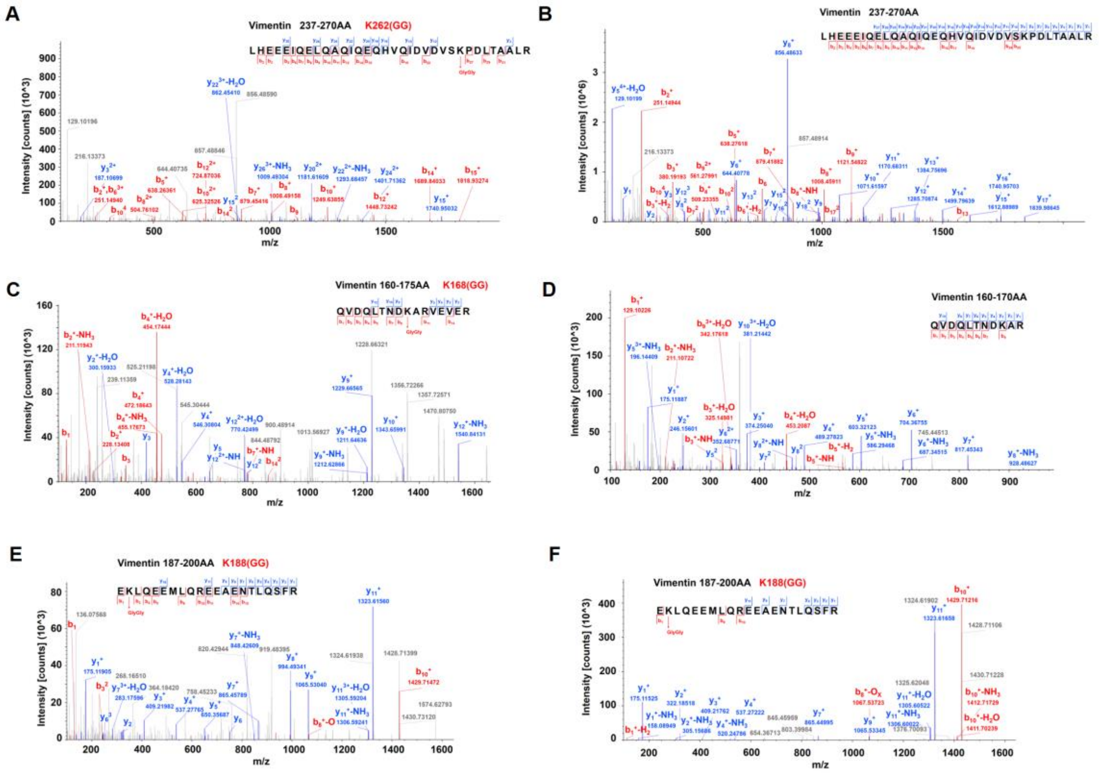

Figure S5. The mutation of K104Q abolish ubiquitination of vimentin at K262 and K168. A and **B**. The secondary mass spectrogram exhibited the ubiquitination of Vimentin K262 in H1299 overexpressing vimentin-WT-Flag (**A**) and vimentin-K104Q-Flag (**B**) cells using co-IP combined with LC–MS/MS. **C** and **D**. The secondary mass spectrogram exhibited the ubiquitination of vimentin K168 in H1299 overexpressing vimentin-WT-Flag (**C**) and vimentin-K104Q-Flag (**D**). **E** and **F**. The secondary mass spectrogram exhibited the ubiquitination of vimentin K188 in H1299 overexpressing vimentin-WT-Flag (**E**) and vimentin-K104Q-Flag (**F**).

Supplemental Figure 6

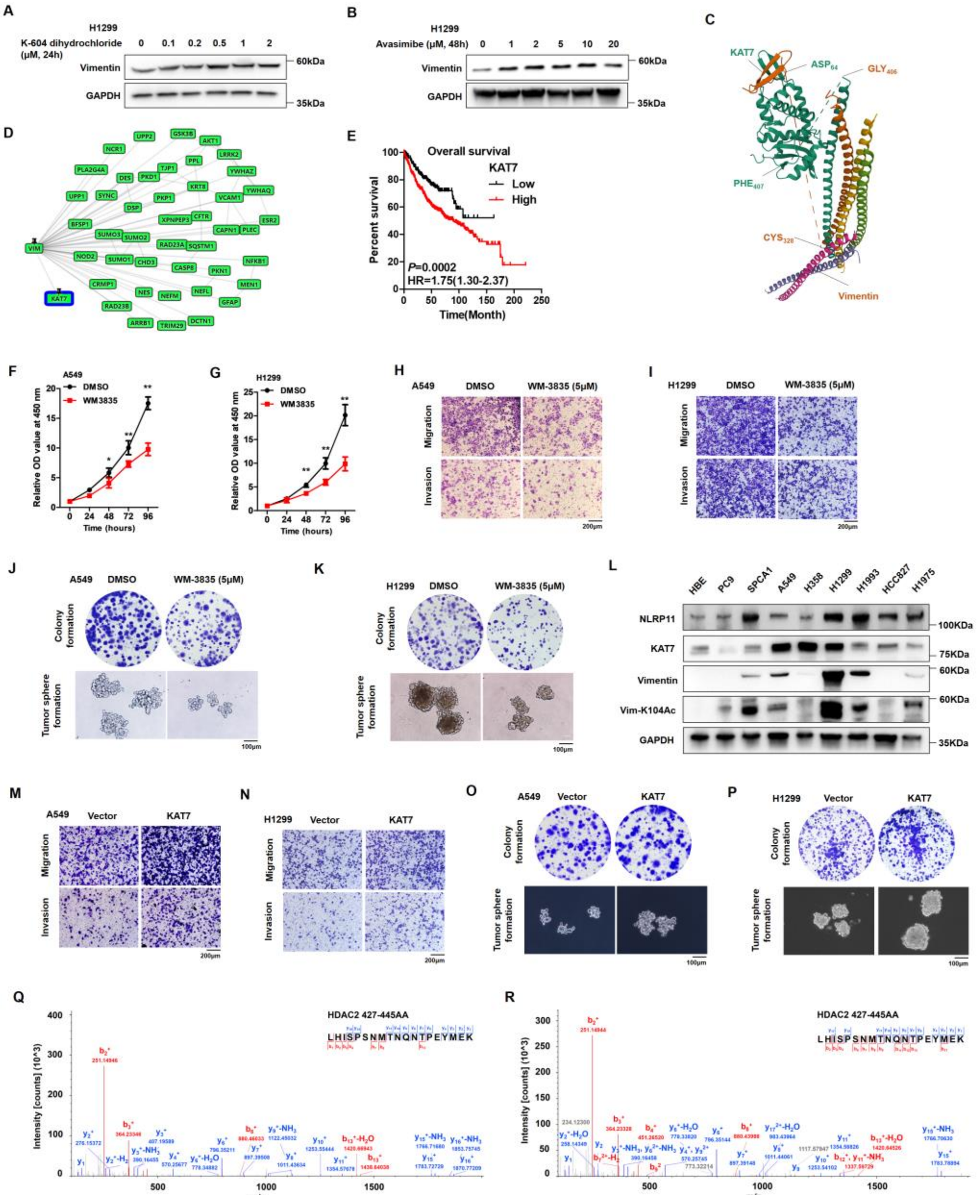

Figure S6. KAT7 promotes the malignant phenotype of LUAD. A and **B**. The effects of an ACAT1 inhibitor (K-604 dihydrochloride) (**A**) and an ACAT2 inhibitor (avasimibe) (**B**) on the expression of vimentin were detected using western blotting. **C**. The binding prediction of KAT7 and vimentin protein were simulated via AutoDock Vina v.1.2.2 molecular docking analysis. **D**. The potential acetyltransferase binding to NLRP11 was analyzed using GPS–Prot Protein–Protein Interactions Analysis (http://gpsprot.org/index.php). **E**. Kaplan–Meier plotter was used to analyze the relationship between the expression of KAT7 and the overall survival of LUAD patients. **F** and **G**. CCK-8 assay was used to evaluate the effect of WM-3835 (5 μM) on the proliferation of A549 (**F**) and H1299 (**G**) cells. **H** and **I**. Transwell invasion and migration assays were used to measure the effects of WM-3835 (5 μM) on metastasis abilities in A549 (**H**) and H1299 (**I**) cells. **J** and **K**. Colony formation and tumor sphere formation were performed to measure the effect of WM-3835 (5 μM) on the clonalities of sphere-forming A549 (**J**) and H1299 (**K**) cells. **L**. The expression of NLRP11, KAT7, vimentin, and vimentin-K104Ac protein between HBE and LUAD cell lines were detected using western blotting. **M** and **N**. Transwell invasion and migration assays were used to detect the effect of KAT7 on the metastatic abilities of A549 (**M**) and H1299 (**N**) cells. **O** and **P**. Colony formation and tumor sphere formation assays were performed to measure the effect of KAT7 on the colony formation of sphere-forming A549 (**O**) and H1299 (**P**) cells. **Q** and **R**. The secondary mass spectrogram exhibited the peptide segments of HDAC2 in H1299 overexpressing vimentin-WT-Flag (**Q**) and vimentin-K104Q-Flag (**R**) cells using co-IP combined with LC–MS/MS. (* $P < 0.05$, ** $P < 0.01$).
