# Supplementary material for: The NLRP11 Protein Bridges the Histone Lysine Acetyltransferase KAT7 to Acetylate Vimentin in the Early Stage of Lung Adenocarcinoma
Source: Adv Sci (Weinh). 2023 Jul 9;10(25):2300971. doi: 10.1002/advs.202300971 (PMC10477884; doi:10.1002/advs.202300971)
Supplement: Supplementary file 2 — Supporting Information [file ADVS-10-2300971-s002.pdf]

ADVANCED SCIENCE
Open Access

Supporting Information

for *Adv. Sci.*, DOI 10.1002/advs.202300971

The NLRP11 Protein Bridges the Histone Lysine Acetyltransferase KAT7 to Acetylate Vimentin in the Early Stage of Lung Adenocarcinoma

Rui Yang, Weilin Peng, Shuai Shi, Xiong Peng, Qidong Cai, Zhenyu Zhao, Boxue He, Guangxu Tu, Wei Yin, Yichuan Chen, Yuqian Zhang, Fang Liu, Xiang Wang, Desheng Xiao and Yongguang Tao**

The NLRP11 protein bridges the histone lysine acetyltransferase KAT7 to acetylate vimentin in the early stage of lung adenocarcinoma

Rui Yang, Weilin Peng, Shuai Shi, Xiong Peng, Qidong Cai, Zhenyu Zhao, Boxue He, Guangxu Tu, Wei Yin, Yichuan Chen, Yuqian Zhang, Liu Fang, Xiang Wang, Desheng Xiao* and Yongguang Tao*

* To whom correspondence should be addressed to Desheng Xiao and Yongguang Tao

Email: xdsh96@csu.edu.cn; taoyong@csu.edu.cn

Supplementary figures

Supplemental Figure 7

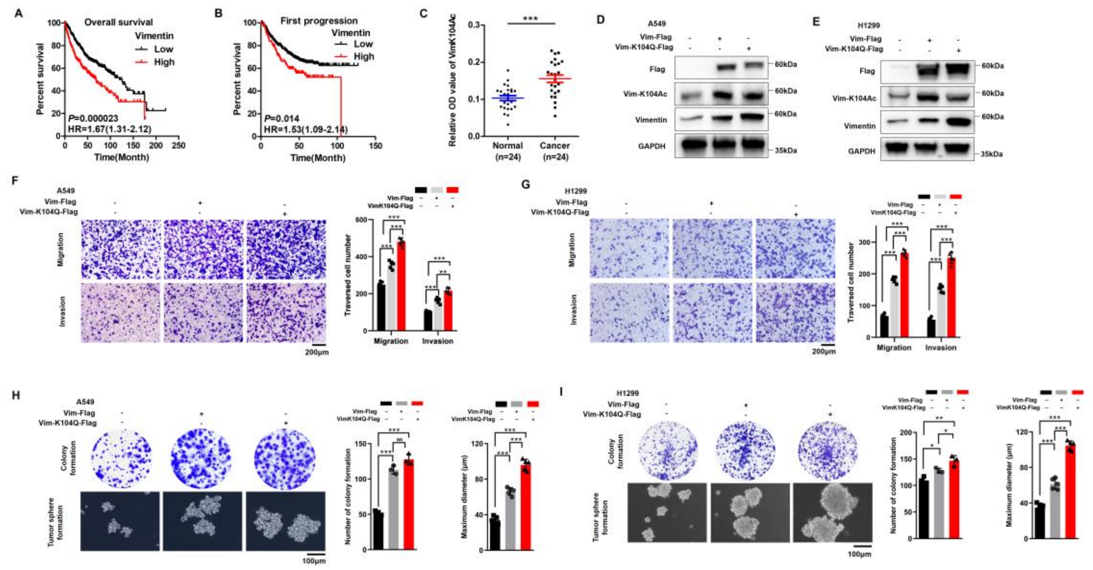

Figure S7. Vimentin-K104Ac promotes the malignant phenotype of LUAD. A and **B**. The relationships between vimentin mRNA expression and the prognosis of LUAD, including OS (**A**) and FP (**B**), were analyzed in gene chips from the Kaplan–Meier plotter database. **C**. ELISA was used to detect the expression of vimentin-K104Ac in serum from patients with LUAD (Cancer) and the Blood Transfusion Department (Normal). **D** and **E**. Western blotting was used to verify A549 (**D**) and H1299 (**E**) cells overexpressing vimentin/vimentin-K104Q and vector cells. **F** and **G**. Transwell invasion and migration assays were used to measure the metastatic abilities of A549 (**F**) and H1299 (**G**) cells overexpressing vimentin/vimentin-K104Ac and vector cells. **H** and **I**. Colony formation and tumor sphere formation assays were used to estimate the clonalities and sphere-forming capability of A549 (**H**) and H1299 (**I**) cells overexpressing vimentin/vimentin-K104Ac and vector cells (* $P < 0.05$, ** $P < 0.01$, *** $P < 0.001$).

Supplemental Figure 8

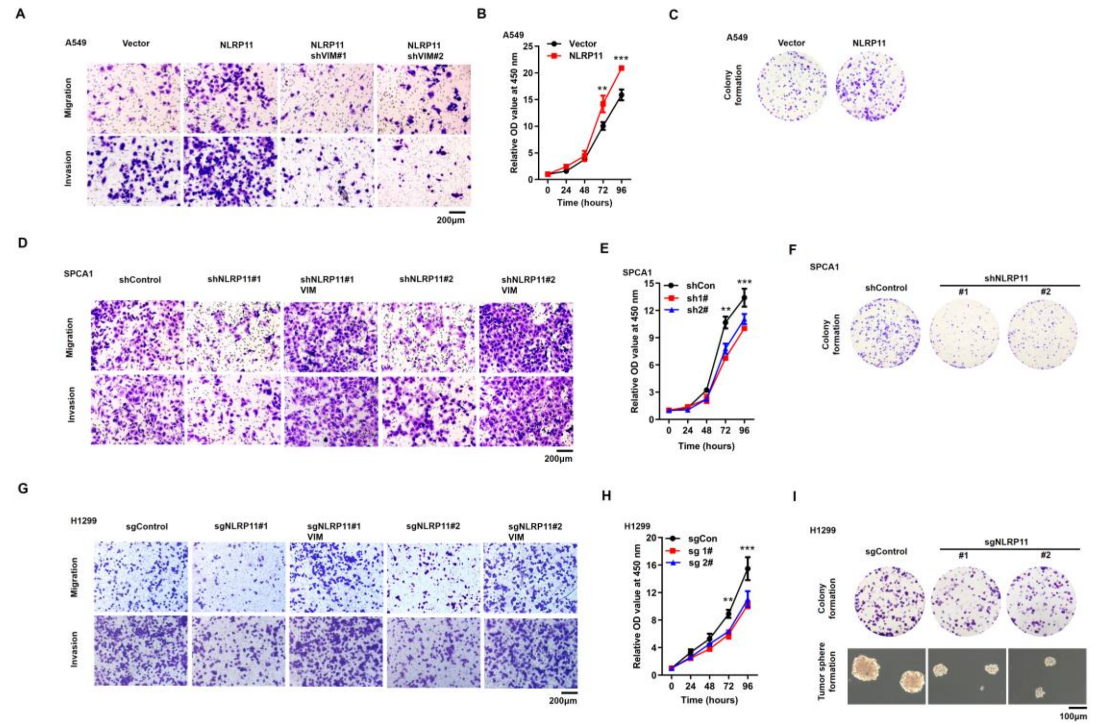

Figure S8. NLRP11 promotes the malignant phenotype of LUAD in vitro. A. The metastatic abilities of A549 cells overexpressing NLRP11 and vimentin knockdown cell lines were measured using Transwell invasion and migration assays. **B**. CCK-8 assays were used to measure proliferation. **C**. Colony formation assays were used to estimate clonalities in A549 NLRP11 cell lines. **D**. The metastatic abilities of SPCA1 shNLRP11 and rescuing vimentin cell lines were measured using Transwell invasion and migration assays. **E**. CCK-8 assays were used to measure proliferation. **F**. Colony formation assays were used to estimate clonalities in SPCA1 shNLRP11 cell lines. **G**. The metastatic abilities of H1299 sgNLRP11 and rescuing vimentin cell lines were measured using Transwell invasion and migration assays. **H**. CCK-8 assays were used to measure proliferation. **I**. Colony formation and tumor sphere formation assays were used to estimate clonalities and sphere formation in H1299 sgNLRP11 cell lines (** $P < 0.01$, *** $P < 0.001$).

Supplemental Figure 9

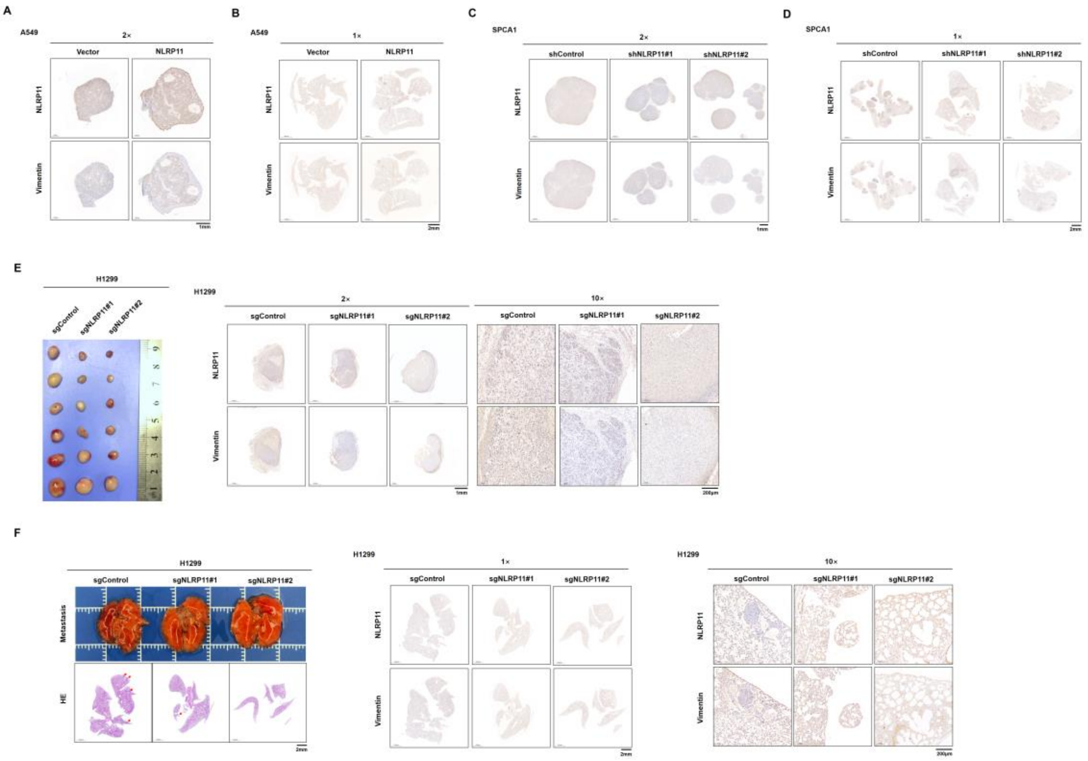

Figure S9. NLRP11 promotes the malignant phenotype of LUAD in vivo. A and **B**. IHC was used to measure the expression of NLRP11 and vimentin in xenograft tumors (**A**) and lung tissues with metastatic tumor cells (**B**) in A549 NLRP11 cell lines. **C** and **D**. IHC was used to measure the expression of NLRP11 and vimentin in xenograft tumors (**C**) and lung tissues with metastatic tumor cells (**D**) in SPCA1 shNLRP11 cell lines. **E** and **F**. Xenograft tumor and experimental metastases via tail vein injection were used to estimate cell proliferation (**E**) and metastatic abilities (**F**) of H1299 sgNLRP11 cell lines in vivo, and IHC was used to measure the expression of NLRP11 and vimentin.

Supplemental Figure 10

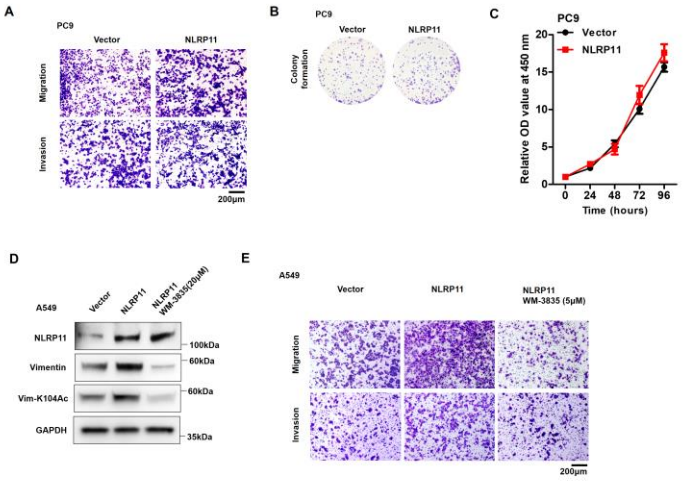

Figure S10. NLRP11 could only increase the malignant phenotype of vimentin-positive (+) LUAD cells via KAT7. A. Transwell invasion and migration assays were used to measure the metastatic abilities of PC9 NLRP11 and vector cells. **B**. Colony formation was used to estimate clonalities in PC9 cells overexpressing NLRP11 and vector cells. **C**. CCK-8 was used to scale the proliferation of PC9 cells overexpressing NLRP11 and vector cells. **D**. The regulatory effect of WM-3835 on the expression of vimentin and vimentin-K104Ac was tested using western blotting in A549 cells overexpressing NLRP11. **E**. The effects of WM-3835 on the metastatic abilities of A549 cells overexpressing NLRP11 were detected using Transwell invasion and migration assays.

Supplemental Figure 11

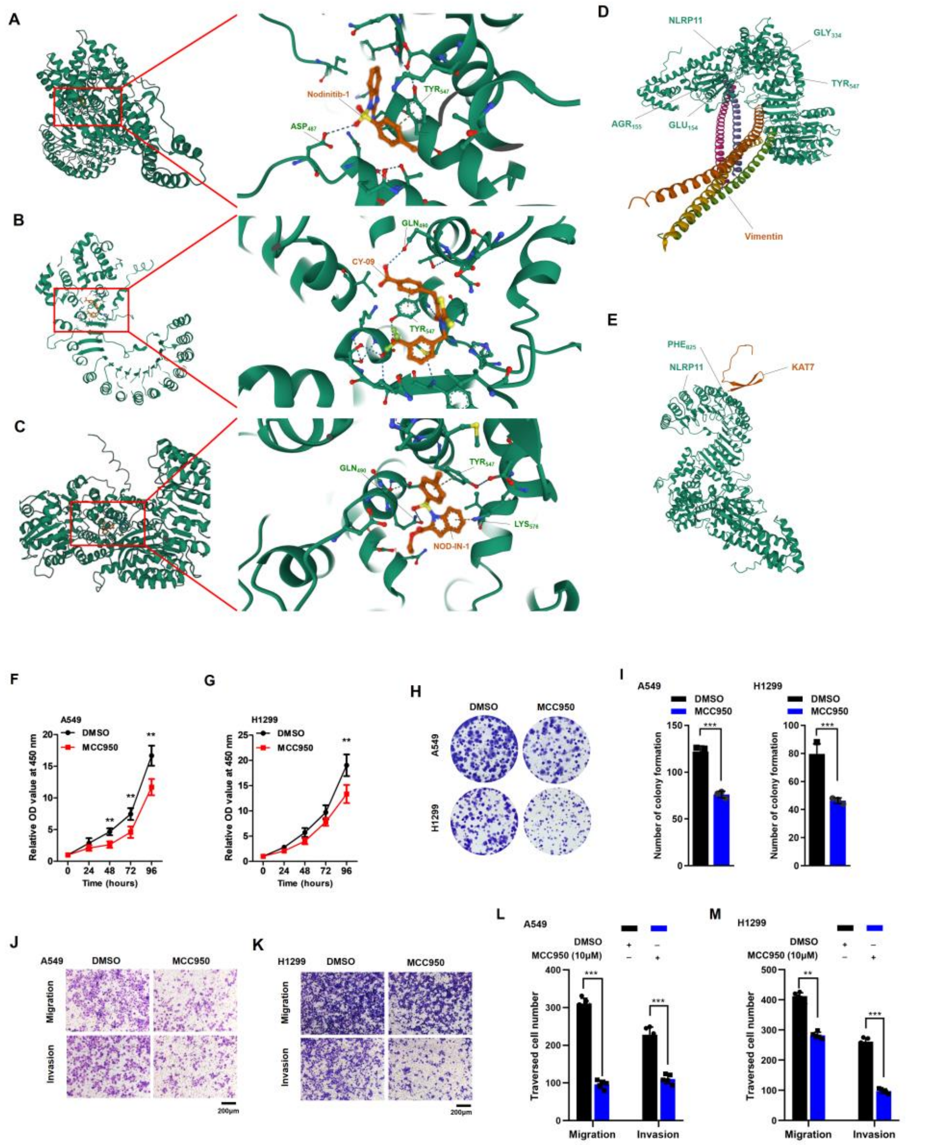

Figure S11. The sieve of NOD-like inhibitors and the bioeffects of MCC950 were performed. A, B, and **C.** The binding affinities and modes of interaction between the drug candidates Nodinitib-1 (**A**), CY-09 (**B**), NOD-IN-1 (**C**), and NLRP11 protein were analyzed using AutoDock Vina v.1.2.2, and the binding energies were −6.703, −7.094, and −6.810 kcal/mol, respectively. **D** and **E**. The prediction of NLRP11 binding to vimentin (**D**) and KAT7 (**E**) protein were analyzed using AutoDock Vina v.1.2.2 molecular docking analysis. **F** and **G**. CCK-8 assays were used to test the effects of MCC950 on the proliferation of A549 (**F**) and H1299 (**G**) cells. **H** and **I**. Colony formation assays were used to assess the clonalities of A549 and H1299 cells treated with MCC950. **J**, **K**, **L,** and **M**. Transwell invasion and migration assays were used to measure the influence of MCC950 on the metastatic abilities of A549 (**J** and **L**) and H1299 (**K** and **M**) cells.
